# Impairment of chaperone-mediated autophagy leads to selective lysosomal degradation defects in the lysosomal storage disease cystinosis

**DOI:** 10.15252/emmm.201404223

**Published:** 2015-01-13

**Authors:** Gennaro Napolitano, Jennifer L Johnson, Jing He, Celine J Rocca, Jlenia Monfregola, Kersi Pestonjamasp, Stephanie Cherqui, Sergio D Catz

**Affiliations:** 1 Department of Molecular and Experimental Medicine, The Scripps Research InstituteLa Jolla, CA, USA; 2 Department of Pediatrics, University of California San DiegoLa Jolla, CA, USA; 3 Cancer Center Microscopy Shared Resource, University of California, San DiegoLa Jolla, CA, USA

**Keywords:** autophagy, CTNS, cystinosis, lysosomal storage disorder, lysosomal trafficking

## Abstract

Metabolite accumulation in lysosomal storage disorders (LSDs) results in impaired cell function and multi-systemic disease. Although substrate reduction and lysosomal overload-decreasing therapies can ameliorate disease progression, the significance of lysosomal overload-independent mechanisms in the development of cellular dysfunction is unknown for most LSDs. Here, we identify a mechanism of impaired chaperone-mediated autophagy (CMA) in cystinosis, a LSD caused by defects in the cystine transporter cystinosin (CTNS) and characterized by cystine lysosomal accumulation. We show that, different from other LSDs, autophagosome number is increased, but macroautophagic flux is not impaired in cystinosis while mTOR activity is not affected. Conversely, the expression and localization of the CMA receptor LAMP2A are abnormal in CTNS-deficient cells and degradation of the CMA substrate GAPDH is defective in *Ctns*^*−/−*^ mice. Importantly, cysteamine treatment, despite decreasing lysosomal overload, did not correct defective CMA in *Ctns*^*−/−*^ mice or LAMP2A mislocalization in cystinotic cells, which was rescued by CTNS expression instead, suggesting that cystinosin is important for CMA activity. In conclusion, CMA impairment contributes to cell malfunction in cystinosis, highlighting the need for treatments complementary to current therapies that are based on decreasing lysosomal overload.

## Introduction

Defects in specific enzymes and transporters cause lysosomal accumulation of metabolites in lysosomal storage disorders (LSDs) (Futerman & van Meer, 2004). This results in impaired cell function, tissue failure and multi-systemic diseases (Futerman & van Meer, 2004; Settembre *et al*, 2013). Importantly, the biological mechanisms that lead from lysosomal enzyme deficiency to cell dysfunction are still largely unknown (Futerman & van Meer, 2004; Settembre *et al*, 2013). Although substrate reduction and lysosomal overload-decreasing therapies ameliorate or delay the development of disease (Cox, 2005; Elliot-Smith *et al*, 2008; Marshall *et al*, 2010), they do not always prevent lysosomal dysfunction, which ultimately leads to cell malfunction and tissue failure, suggesting that substrate accumulation is not the only mediator of toxicity. A striking example of this is represented by cystinosis, a LSD resulting from defects in the lysosomal transporter of cystine, cystinosin (CTNS) (Town *et al*, 1998; Cherqui *et al*, 2001; Kalatzis *et al*, 2001; Taranta *et al*, 2010). Cystinosis is characterized by defects in several tissues, especially the kidneys and eyes, but also affects other organs (Nesterova & Gahl, 2008). In cystinosis, lysosomal overload-decreasing therapy is achieved by the use of cysteamine, a drug that reduces intralysosomal levels of cystine and that is the current treatment of choice for cystinotic patients (Gahl *et al*, 2007). The efficiency of cysteamine in retarding the rate of glomerular deterioration and improvement of linear growth in children with cystinosis (da Silva *et al*, 1985; Gahl *et al*, 2002) demonstrates the effectiveness of cystine-depleting therapies. However, renal injury is not observed in all patients with cystinosis (Anikster *et al*, 1999). In addition, although delayed, progressive renal injury occurs despite cystine depletion therapy in patients with nephropathic cystinosis (Cherqui, 2012). Therefore, it is generally accepted that cystine accumulation may not be the only cause for all the defects observed in cystinosis (Vaisbich *et al*, 2011; Cherqui, 2012). Thus, to improve treatment of cystinosis and other LSDs, it is crucial to understand the defective molecular mechanisms that could lead to cell dysfunction and tissue injury.

Impairment in lysosomal function in different LSDs has been associated with macroautophagy defects (Settembre *et al*, 2008a,b); however, it is not clear whether macroautophagy defects are common to all LSDs, and a mechanistic analysis of other autophagic pathways in LSDs is missing. Autophagy is an essential cellular process that consists of the digestion of cytoplasmic components through lysosomal degradation (Klionsky, 2007). Autophagic pathways represent a major protective mechanism that, in addition to their role in providing cells with amino acids and nutrients, also allow cell survival in response to multiple stressors, including starvation and oxidative stress, and help defend organisms against degenerative and neoplastic diseases (Levine & Kroemer, 2008; Martinou & Kroemer, 2009). Several autophagic pathways have been described, including macroautophagy, microautophagy and chaperone-mediated autophagy (CMA), which differ in their regulation and selectivity. For instance, macroautophagy is a degradative process by which bulk cytoplasmic components and organelles are sequestered in a structure known as the autophagosome and digested upon its fusion to the lysosome (He & Klionsky, 2009). Autophagosome formation, fusion to lysosomes and degradation of the autophagic content is a highly regulated process whose rate is determined by different factors, such as nutrient availability and cell stress (He & Klionsky, 2009). Different from macroautophagy, CMA is a selective form of autophagy through which single cytoplasmic proteins containing a recognition motif (the pentapeptide KFERQ) are bound by a cytoplasmic chaperone complex and delivered to a lysosomal receptor, which mediates the translocation of the CMA substrates into the lysosomal lumen, allowing their degradation (Kaushik & Cuervo, 2012). Although selective, CMA accounts for the digestion of 30% of the proteome (Kaushik & Cuervo, 2012). To date, the only known lysosomal receptor for CMA is lysosome-associated membrane protein 2A (LAMP2A), one of the three variants encoded by the *Lamp2* gene. LAMP2A is the only isoform required for CMA function (Cuervo & Dice, 2000b; Massey *et al*, 2006a) but is dispensable for other types of autophagy (Cuervo & Dice, 1996, 2000b; Massey *et al*, 2006a). Substrate binding to LAMP2A is an essential step for CMA, and cells use changes in LAMP2A levels to upregulate or downregulate CMA (Cuervo & Dice, 2000a,b; Massey *et al*, 2006a). Changes in the rate of LAMP2A synthesis, its regulated degradation at the lysosomal membrane and its sub-compartmentalization to this organelle, all contribute to modulate CMA activity (Kaushik & Cuervo, 2012). Autophagic pathways are closely interconnected (Park & Cuervo, 2013), and although they are preferentially activated in a time- and stimuli-specific manner, most cells activate macroautophagy in response to blockage of CMA and vice versa (Massey *et al*, 2006a; Kaushik *et al*, 2008). Despite this bidirectional compensation, these pathways are not redundant. Importantly, primary defects in CMA have been linked to human diseases, including neurodegenerative disorders and nephropathies (Bejarano & Cuervo, 2010), indicating that compensatory activation of macroautophagy is not sufficient to fully compensate for CMA defects.

Although abnormal accumulation of autophagosome markers has been shown in cystinotic cells (Sansanwal *et al*, 2010), a mechanistic analysis of autophagic pathways in cystinosis is lacking. In this report, we show that, different from other LSDs, cells and tissues lacking CTNS expression are characterized by increased autophagosome numbers, but functional macroautophagic flux. We also show abnormal expression levels and localization of the CMA receptor LAMP2A in cystinotic cells and defective lysosomal degradation of the CMA substrate GAPDH in cystinotic lysosomes, which was independent from cystine accumulation and lysosomal overload. Our data highlight that CMA impairment is an important contributor to the pathogenesis of cystinosis and underline the need for treatments complementary to substrate depletion therapies in cystinosis.

## Results

### CTNS-deficient cells and tissues show an increased number of autophagosomes

**Figure 1:**
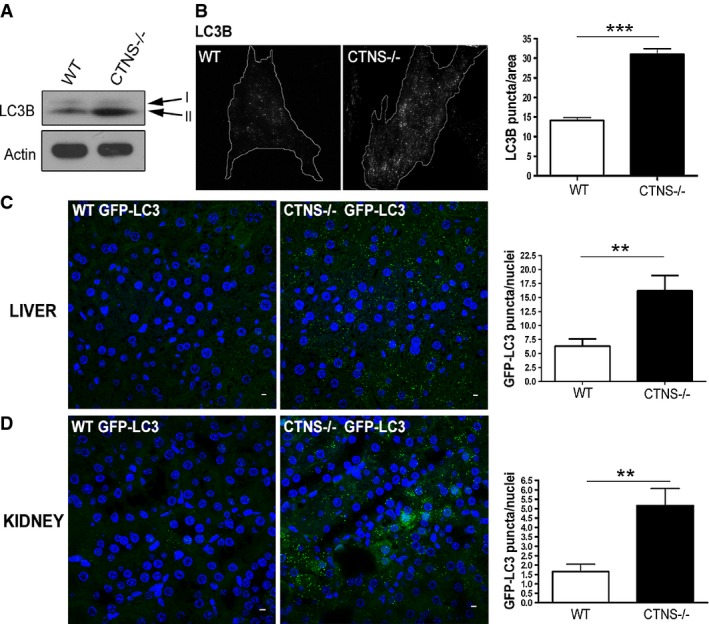
CTNS-deficient cells and tissues have increased number of autophagosomes Expression levels of the autophagosome marker LC3B-II were evaluated in wild-type (WT) and *Ctns*^*−/−*^ fibroblasts by Western blot (WB). Data are representative of five different experiments with similar results.

Abnormal lysosomal function in LSDs has been associated with macroautophagy impairment, due to defective lysosomal fusion with autophagosomes or impaired degradation of autophagy substrates (Settembre *et al*, 2008a,b). In this work, to better understand the regulation of autophagy in cystinosis, we analyze macroautophagic flux and chaperone-mediated autophagy using *Ctns*^*−*/*−*^ mice, *Ctns*^*−*/*−*^-GFP-LC3 transgenic mice and *Ctns*^*−*/*−*^-derived neonatal skin fibroblasts. First, to assess macroautophagy in cystinotic cells, we evaluated the expression levels of LC3B-II, a protein known to specifically associate with autophagosomes and rarely with other vesicular structures (Owen *et al*, 2014) and whose levels correlate with the number of autophagosomes (Kabeya *et al*, 2000; Klionsky *et al*, 2012). As shown in Fig1A, the levels of LC3B-II were highly increased in CTNS-deficient fibroblasts, indicating that the number of autophagosomes was increased in these cells. The same results were confirmed by immunofluorescence (IF), which revealed increased number of endogenous LC3B-positive structures in CTNS-deficient fibroblasts (Fig1B).

WT and *Ctns*^*−/−*^ fibroblasts were stained using LC3B antibodies and analyzed by fluorescence microscopy. The number of LC3B-positive puncta was quantified by the ImagePro software and normalized per area arbitrary units. Results are mean ± SEM (*n *= 27 WT and 24 *Ctns*^*−/−*^ cells). ***P < 0.001 (unpaired *t*-test).

Immunofluorescence analysis of liver (C) and kidney (D) tissues from WT and *Ctns*^*−/−*^ GFP-LC3 transgenic mice using anti-GFP antibodies. Quantification of LC3 puncta was obtained by counting the number of GFP-positive structures relative to the number of nuclei in the same field, using the ImagePro software. GFP-LC3 puncta were counted by analyzing 6 to 10 fields per tissue section (200–400 nuclei per field), in a total of 3 WT and 3 *Ctns*^*−/−*^ mice expressing GFP-LC3. Results are mean ± SEM. In (C), **P = 0.0029; in (D), **P = 0.0025 (unpaired *t*-test). Scale bar: 5 μm.

To evaluate whether the number of autophagosomes was also increased *in vivo*, we crossed WT and *Ctns*^*−/−*^ mice with a transgenic strain expressing GFP-LC3 (Mizushima *et al*, 2004) (see Materials and Methods). Confocal microscopy analysis of liver and kidney sections showed that the number of GFP-positive structures was highly increased in *Ctns*^*−/−*^ mouse liver (Fig1C) and kidney (Fig1D) tissues compared to WT, confirming an increased number of autophagosomes in CTNS-deficient mice.

### Maturation of autophagosomes is not impaired in CTNS-deficient cells

**Figure 2:**
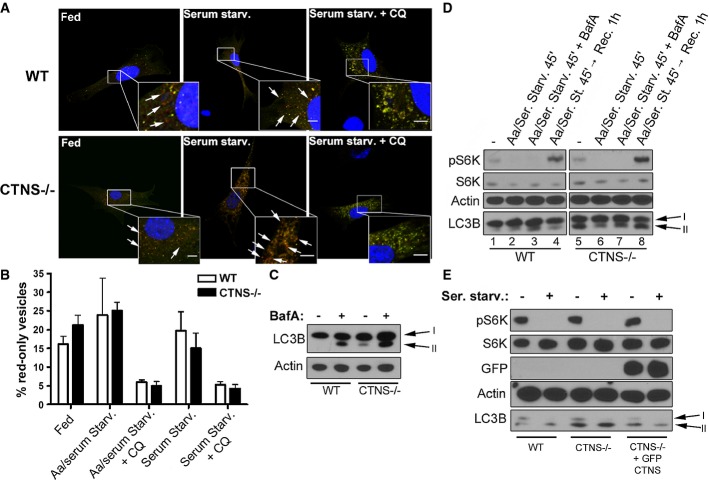
Analysis of the macroautophagic flux in *Ctns*^*−*/*−*^ cells Representative images of wild-type (WT) and *Ctns*^*−/−*^ mouse fibroblasts transfected with the ptfLC3 vector under resting conditions (fed), or after serum starvation (Serum Starv.) in the presence or absence of the alkalinizing drug chloroquine (CQ). GFP and RFP staining was analyzed by confocal microscopy. Examples of red-only puncta (mature autophagosomes) are indicated with arrows. Scale bar: 5 μm.

The autophagic flux is a dynamic process that involves autophagosomes formation and their subsequent fusion to lysosomes for digestion of the autophagic content (He & Klionsky, 2009). To analyze whether the increased number of autophagosomes found in *Ctns*^*−/−*^ cells and tissues is caused by an accumulation of autophagosomes due to their impaired maturation, we used the following approaches: first, we evaluated whether fusion of autophagosomes with lysosomes was impaired in *Ctns*^*−/−*^ cells. To this end, we used the tandem fluorescently tagged RFP-GFP-LC3 (ptfLC3), in which LC3 is expressed as a fusion protein with both GFP and RFP in tandem (Kimura *et al*, 2007). This assay is based on the nature of the GFP protein, which is highly sensitive to and loses fluorescence in the acidic environment of the lysosomes, whereas RFP is not. Thus, in the non-acidic environment of autophagosomes, before fusion with lysosomes, LC3 puncta show both GFP and RFP signals, but once the maturation to autophagolysosomes occurs, they exhibit the RFP signal only (Kimura *et al*, 2007). Of note, no difference in intralysosomal pH has been described between WT and *Ctns*^*−/−*^ fibroblasts (Oude Elferink *et al*, 1983). WT and *Ctns*^*−/−*^ fibroblasts were transfected with the ptfLC3 vector, and the autophagic flux was induced by starvation. Confocal microscopy analysis revealed that, although the total number of GFP/RFP-positive structures was increased in *Ctns*^*−/−*^ cells, the percent of RFP-only-positive structures was similar in both WT- and *Ctns*^*−/−*^-transfected fibroblasts (Fig2A and B), indicating that fusion of autophagosomes with lysosomes was similar in these cells for all conditions tested. As a control, chloroquine (CQ) treatment, which increases lysosomal pH (Klionsky *et al*, 2012), inhibited GFP quenching and proteolysis in both WT and *Ctns*^*−/−*^ fibroblasts (Fig2A and B).

The percentage of mature autophagosomes (red-only vesicles) was calculated based on the ratio between the number of red-only puncta and the total number of autophagosomes (number of green and red + red-only puncta). The graph is representative of three different experiments with similar results. Results are mean ± SEM (*n *= 7 cells per condition). In addition to the experimental conditions shown in (A), the Aa/serum starvation condition is included.

WT and *Ctns*^*−/−*^ fibroblasts under resting conditions were treated with 100 nM bafilomycin A (BafA) for 2 h and LC3B-II levels were analyzed by Western blot (WB).

Phosphorylation levels of the mTOR complex kinase 1 substrate S6K and LC3B-II levels in WT and *Ctns*^*−/−*^ fibroblasts were measured by WB under resting conditions (−), withdrawal of both amino acids and serum (Aa/Ser. Starv.) and subsequent recovery by replacement of starvation medium with normal cell growth medium (Rec), in the presence or absence of 100 nM BafA for the indicated time.

WB analysis showing phosphorylation levels of the mTOR substrate S6K and expression of LC3B-II in resting or serum-starved WT,*Ctns*^*−/−*^ and *Ctns*^*−/−*^ fibroblasts expressing GFP-CTNS. Source data are available online for this figure.

**Figure 3:**
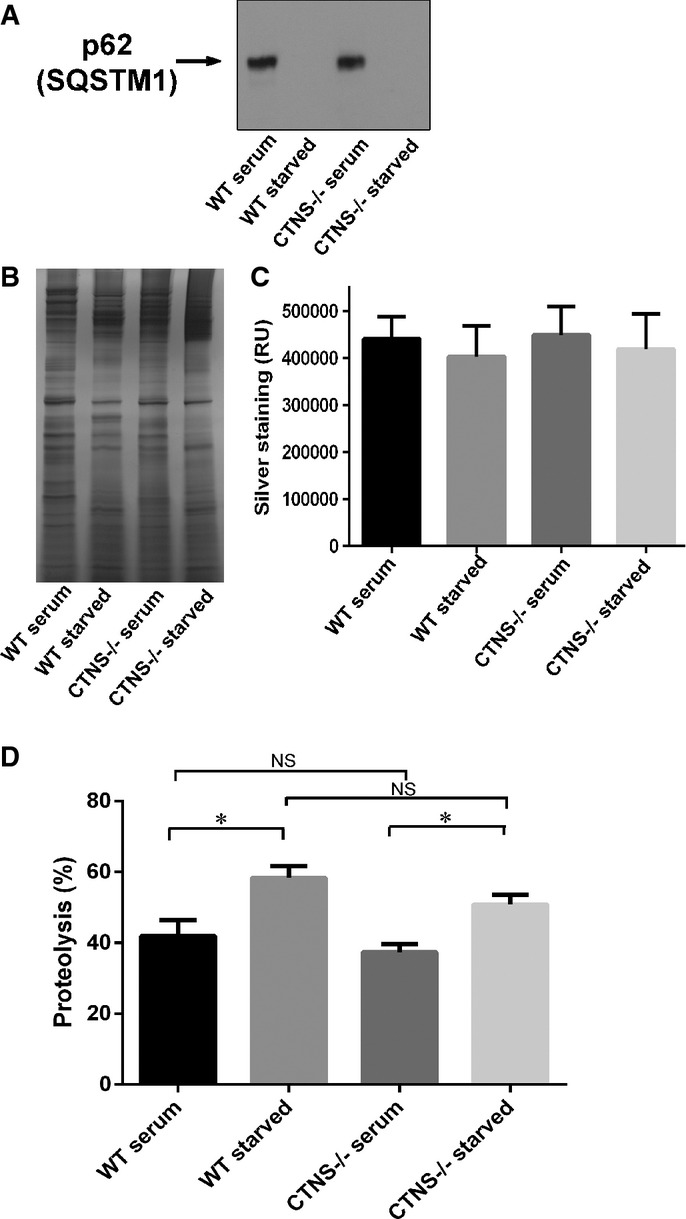
Degradation of SQSTM1/p62 and long-lived proteins is not impaired in CTNS-deficient cells Analysis of the expression of endogenous SQSTM1 in wild-type or *Ctns*^*−*/*−*^ mouse fibroblasts under fed (serum) or starved (15 h serum starvation) conditions. In these assays, 20 μg of total protein lysates were resolved by SDS-PAGE and analyzed by Western blot (WB; A). Equal loading was established by silver staining (B). Quantification (mean ± SEM) of five different areas of each silver-stained lane are shown in (C).

Second, we examined autophagic flux by the LC3 turnover assay, which measures autophagosome maturation and lysosomal digest-ion of the autophagic content by comparing LC3-II amounts in the presence or absence of bafilomycin A (BafA), a drug that increases lysosomal pH and also blocks lysosomal fusion (Tanida *et al*, 2005). Because LC3-II is present in both outer and inner autophagosome membranes, LC3-II itself is degraded after lysosomal fusion (Tanida *et al*, 2005) Thus, while cells with functional autophagosomal maturation show increased levels of LC3-II upon BafA treatment, cells characterized by impaired autophagosome maturation show no change in LC3-II levels after treatment with BafA. As shown in Fig2C, BafA treatment of resting (non-starved) WT and *Ctns*^*−/−*^ fibroblasts caused accumulation of LC3B-II in both cell types, indicating that *Ctns*^*−/−*^ cells have functional fusion and degradation of the autophagic content. In addition, LC3B-II levels in BafA-treated *Ctns*^*−/−*^ cells were higher than those seen in BafA-treated WT cells (Fig2C), also indicating that synthesis of new autophagosomes is not defective in *Ctns*^*−/−*^ cells and that increased levels of LC3B-II in these cells result from an increased and active basal autophagosomal formation. These results were further confirmed when LC3B-II levels were analyzed under different conditions of starvation, which stimulates autophagic flux by increasing both synthesis and degradation of autophagosomes, and that can result in either increase or decrease in LC3B-II levels depending on the cell type and on the rate of the basal autophagic flux (Klionsky *et al*, 2012). Withdrawal of both amino acids and serum for a short incubation time (45 min), which represents an acute starvation protocol (complete nutrient deprivation) and increases the rate of fusion and degradation of autophagosomes due to immediate nutrient needs, induced LC3B-II degradation in *Ctns*^*−/−*^ fibroblasts (Fig2D, lane 6, bottom panel), which was suppressed by BafA treatment (Fig2D, lane 7, bottom panel), indicating also in this case that autophagosome maturation and the macroautophagy response to stress conditions are functional in *Ctns*^*−/−*^ cells (see also Supplementary Fig S1A, lanes 6, 7 and 8). In addition, replacement of starvation medium with normal cell growth medium restored LC3B-II levels to basal conditions (Fig2D, compare lane 8 with lane 5), suggesting also in this case that synthesis of new autophagosomes is not impaired in cystinotic cells. Although the greater accumulation of LC3B-II observed after nutrient recovery in *Ctns*^*−/−*^ cells compared to wild-type cells (Fig2D, lanes 4 and 8) may suggest that degradation is slower in *Ctns*^*−/−*^ cells under recovery experimental conditions, the observation that starvation itself induces a prompt reduction in the levels of LC3B-II in *Ctns*^*−*/*−*^ cells (Fig2D lanes 5 and 6, Supplementary Fig S1A and B lanes 6 and 7) argues against a marked defective degradation phenotype in these cells (also shown in Fig3). Finally, milder starvation of *Ctns*^*−/−*^ cells by removal of serum-only for a longer incubation time (5 h), a condition in which increased degradation of autophagosomes is balanced by increased autophagosome formation, caused a mild increase in LC3B-II levels (Supplementary Fig S1A, lane 9), which was strongly exacerbated by BafA treatment (Supplementary Fig S1A, lane 10), also indicating that autophagosomes undergo proper maturation in cystinotic cells. Similar results were obtained when LC3B-II turnover was measured in bone marrow-derived macrophages (BMDM) from WT and *Ctns*^*−/−*^ mice (Supplementary Fig S1B). Altogether, these data show that autophagosome maturation is not impaired in cystinotic cells and that *Ctns*^*−*/*−*^ cells are characterized by increased numbers of autophagosomes but a fully functional autophagic flux.

Degradation of long-lived proteins using metabolic labeling was performed as described in Supplementary Materials and Methods. The data represent the mean ± SEM of nine independent samples analyzed in three independent experiments. *P = 0.009 for WT and *P = 0.0013 for *Ctns*^*−*/*−*^, respectively. NS, not significant (unpaired *t*-test). Source data are available online for this figure.

### Increased autophagosome number in cystinosis is not caused by aberrant mTOR activity

The mammalian target of rapamycin **(**mTOR) signaling pathway represents the major regulatory hub at the interface between cell growth and starvation (Zoncu *et al*, 2011). Amino acids and cell nutrients can activate the mTOR kinase complex, whose activity is responsible for phosphorylation of different substrates important in the promotion of cell growth and suppression of autophagy (Zoncu *et al*, 2011). Conversely, starvation inactivates mTOR, thereby inhibiting anabolic processes and liberating nutrient reserves by activating autophagy.

To determine whether increased autophagosome number in cystinotic cells was due to aberrant mTOR activity, we analyzed the phosphorylation levels of the mTOR substrate p70-S6 kinase (S6K), both in fed and starved WT and *Ctns*^*−/−*^ fibroblasts. This assay showed that the levels of pS6K were comparable in non-starved WT and *Ctns*^*−/−*^ mouse fibroblasts (Fig2D, lanes 1 and 5, upper panel). Furthermore, removal of both amino acids and serum induced loss of mTOR activity in both WT and *Ctns*^*−/−*^ cells (Fig2D, lanes 2 and 6, upper panel), and this activity was restored at similar levels in both cell types by subsequent addition of nutrients (Fig2D, lanes 4 and 8, upper panel), suggesting that the mTOR pathway is functional in cystinotic cells. Finally, lentiviral transduction of *Ctns*^*−/−*^ cells with GFP-CTNS was able to rescue normal LC3 levels without inducing significant changes in mTOR activity (Fig2E), suggesting that increased autophagosome number in cystinotic cells was not caused by aberrant mTOR activity.

### SQSTM1/p62 and long-lived proteins degradation are not impaired in *Ctns*^*−*/*−*^ cells

In order to further evaluate autophagic flux in *Ctns*^*−*/*−*^ cells, we analyzed the degradation of the autophagic flux marker sequestosome 1 (SQSTM1/p62), a ubiquitin-binding scaffold protein that binds to LC3 and GABARAP proteins, is degraded by autophagy and guides ubiquitinated proteins to the autophagic machinery for degradation in the lysosome (Fig3) (Bjorkoy *et al*, 2009). Of note, because long-term serum starvation induces an alteration in the expression of many proteins including commonly used house-keeping proteins (Schmittgen & Zakrajsek, 2000), we established equal loading by silver staining of the same samples run in parallel in SDS-gels (Fig3B). For quantification, five different areas of the silver-stained gel were used for each lane (Fig3C). As shown in Fig3A, starvation induced degradation of SQSTM1/p62 in both WT and *Ctns*^*−*/*−*^ fibroblasts further supporting that autophagic flux is not impaired in cystinosis. This was further confirmed by analysis of long-lived protein degradation using radiolabeled amino acids (Fig3D), which showed similar levels of proteolysis for both WT and *Ctns*^*−*/*−*^ cells. Overall, our data from Figs2 and 3 rule out major defects in autophagosome maturation and degradation in *Ctns*^*−*/*−*^ cells despite increased autophagosome number, which was not caused by aberrant mTOR activity.

### The chaperone-mediated autophagy receptor LAMP2A is downregulated in cystinotic cells

**Figure 4:**
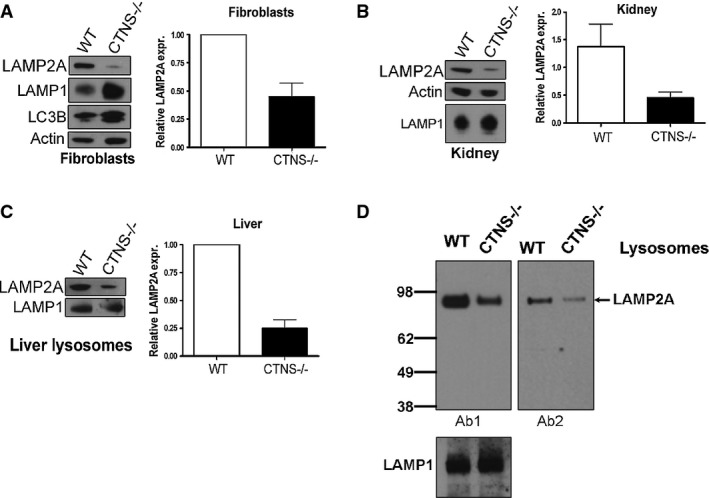
The CMA receptor LAMP2A is downregulated in CTNS-deficient cells The expression levels of the endogenous LAMP2A and LAMP1 proteins were analyzed in wild-type (WT) and *Ctns*^*−/−*^ mouse fibroblasts (A), kidney (B) and liver lysosomes (C) by Western blot. Quantitative densitometry analysis of immunoblots obtained from different experiments was performed by calculating the ratio between LAMP2A and actin signals in each lane. Results are mean ± SEM of four independent experiments with mouse fibroblasts, 5 WT and 6 *Ctns*^*−/−*^ mouse kidneys, 3 WT and 3 *Ctns*^*−/−*^ mouse livers (lysosomal extracts).

Because CMA impairment induces compensatory activation of other autophagic pathways (Massey *et al*, 2006a; Kaushik & Cuervo, 2012), we reasoned that the increase in the rate of macroautophagy could be caused by defects in CMA activity. Therefore, we analyzed protein levels and localization of the CMA receptor LAMP2A, the main regulatory factor of CMA activity. Cystinotic cells show increased size and number of lysosomes as previously detected by electron microscopy and lysosomal volume quantification by stereology (Johnson *et al*, 2013), and also show increased LysoTracker staining measured by FACS (Supplementary Fig S2A), increased LAMP1 staining measured by IF (Supplementary Fig S2B) and increased LAMP1 expression measured by Western blot (Fig4A). Despite the increased lysosomal number, the expression levels of LAMP2A were decreased in *Ctns*^*−/−*^ fibroblasts (Fig4A), mouse kidney (Fig4B), and in lysosomes purified from mouse liver (Fig4C). Finally, *Ctns*^*−*/*−*^ cells showed decreased LAMP2A lysosomal expression when tested using two independent anti-LAMP2A antibodies raised against the carboxyterminal domain of LAMP2A (Fig4D). This includes a commercially available antibody raised against human LAMP2A that has high reactivity to mouse LAMP2A (Ab18528) and an antibody raised against the 12 amino acid cytosolic tail of rat LAMP2A which is identical to mouse protein (GLKRHHTGYEQF), and has largely been demonstrated to detect mouse LAMP2A but not other LAMP2 isoforms (Cuervo & Dice, 1996; Cuervo & Dice, 2000b). Both antibodies recognized the expected band of ∽95 kDa and showed significant decreased expression in cystinotic lysosomes (Fig4D). Next, to determine whether the defective LAMP2A expression in *Ctns*^*−/−*^ cells was caused by impaired transcriptional regulation of LAMP2A, we performed quantitative RT–PCR using LAMP2A-specific primers (see Materials and Methods). Our data revealed that RNA levels of *LAMP2A* (Ref Seq NM_001017959) were similar in fed and starved WT and *Ctns*^*−/−*^ cells (Supplementary Fig S3).

Comparative analysis of the amount of LAMP2A in wild-type or *Ctns*^*−/−*^ liver lysosomes was performed using two different anti-LAMP2A antibodies. 15 μg of lysosomal proteins were loaded in each lane. Ab1, anti-LAMP2A antibody raised against the twelve amino acids of the cytosolic region of rat LAMP2A largely validated to recognize mouse LAMP2A but not other LAMP2 isoforms (Cuervo & Dice, 1996). Ab2, anti-LAMP2A Abcam Ab18528. Decreased lysosomal LAMP2A was evident using either antibody. LAMP1 was used as a loading control. Representative of two independent experiments with similar results. Source data are available online for this figure.

### LAMP2A is mislocalized in cystinotic cells

**Figure 5:**
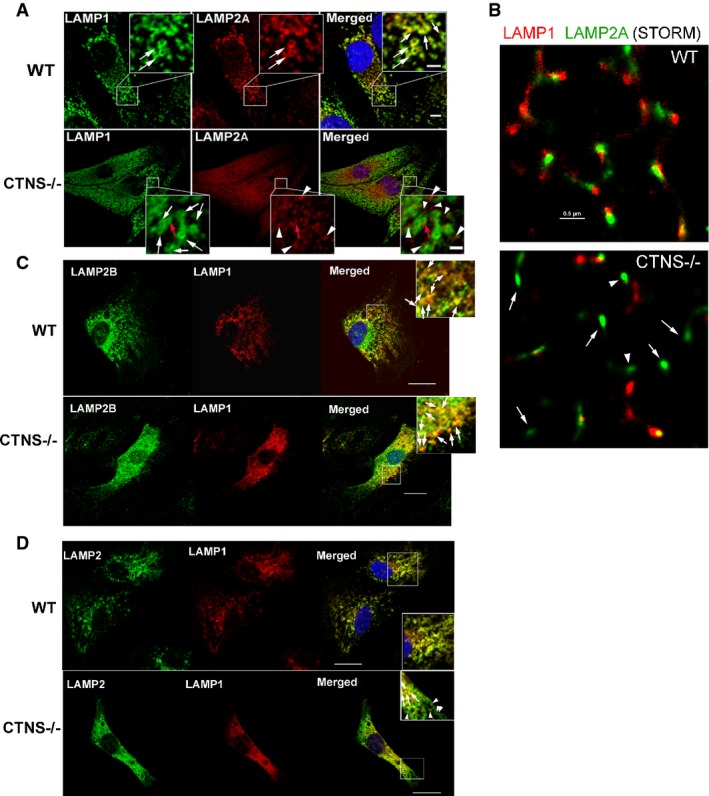
The CMA receptor LAMP2A is mislocalized in CTNS-deficient cells Immunofluorescence analyses of endogenous LAMP1, LAMP2A (A, B), LAMP2B (C) and total LAMP2 (D) localization in wild-type (WT) and *Ctns*^*−/−*^ mouse fibroblasts. (A) Confocal microscopy analysis. White arrows represent LAMP1-positive circular structures (lysosomes) which were visible in both WT and *Ctns*^*−/−*^ cells. Colocalization of LAMP1 and LAMP2A was observed in WT fibroblasts (white arrows, merged panel) but rarely in *Ctns*^*−/−*^ cells (red arrow). Most LAMP2A showed a punctate pattern and lack of colocalization with LAMP1 in *Ctns*^*−/−*^ cells (arrowheads, lower panels). Scale bars: 5 μm. Inset scale bars: 2 μm. (B) High-resolution stochastic optical reconstruction microscopy (STORM) analysis of the localization of LAMP1 and LAMP2A in wild-type and *Ctns*^*−/−*^ cells. In *Ctns*^*−*/*−*^ cells, LAMP2A was detected near (arrowheads, estimated distance > 50 nm) but not always adjacent (10–50 nm) to LAMP1 or at LAMP1-negative structures (arrows). Scale bar: 0.5 μm. (C) Immunofluorescence showing colocalization of endogenous LAMP1 and LAMP2B in both wild-type and CTNS-deficient fibroblasts (arrows). Scale bar: 20 μm. (D) Immunofluorescence analysis of LAMP1 and total LAMP2 (antibody ABL93 DSHB). Arrows show colocalization. Arrowheads show LAMP2-positive LAMP1-negative vesicles.

To better understand the defects affecting LAMP2A protein in *Ctns*^*−/−*^ cells, we analyzed the distribution of endogenous LAMP2A by immunofluorescence analysis by confocal microscopy. This analysis revealed an abnormal localization of LAMP2A in *Ctns*^*−/−*^ cells (Fig5). Thus, different from WT mouse fibroblasts where LAMP2A localized to LAMP1-positive structures resembling lysosomes (Fig 5A, white arrows), LAMP2A localized at puncta distributed in close proximity to but different from LAMP1-positive lysosomes in *Ctns*^*−/−*^ cells (Fig5A, arrowheads) with just a few lysosomes containing LAMP2A being identified (Fig5A, red arrow). To analyze the distribution of LAMP2A in *Ctns*^*−*/*−*^ cells in further detail, we used high-resolution direct stochastic optical reconstruction microscopy (STORM). Using this approach, LAMP2A was observed adjacent (∽20–50 nm distance, STORM lateral resolution limit when detecting endogenous proteins using antibodies) to LAMP1 in wild-type cells, suggesting that they are distributed at common lysosomal microdomains. However, in *Ctns*^*−*/*−*^ cells, LAMP2A was mostly distributed at LAMP1-negative structures or in the proximity (distance >50 nm) but not adjacent to LAMP1, differently from the distribution observed for wild-type cells. Altogether, confocal and STORM analyses suggest that in *Ctns*^*−*/*−*^ cells LAMP2A is either present at non-lysosomal structures (Fig5A) or if at the lysosomal membrane, LAMP2A and LAMP1 appear partially segregated in distinct microdomains (Fig5B).

As a control, we analyzed the localization of LAMP2B in *Ctns*^*−/−*^ and WT cells. Our data show that LAMP2B colocalizes with LAMP1 at lysosomes in both *Ctns*^*−*/*−*^ and WT cells (Fig5C). Next, we analyzed the distribution of total LAMP2 in relationship to that of LAMP1 using the antibody ABL93 (Fig5D). In these studies, we observed full colocalization of LAMP2 with LAMP1 in WT cells and partial localization of LAMP2 with LAMP1 in *Ctns*^*−*/*−*^ cells. This correlates with the lower expression and mislocalization of LAMP2A and with the correct localization of LAMP2B in *Ctns*^*−*/*−*^ cells. Thus, the subpopulation of LAMP2-positive but LAMP1-negative structures observed only in *Ctns*^*−*/*−*^ cells further supports that LAMP2A but not LAMP2B is mislocalized in these cells.

**Figure 6:**
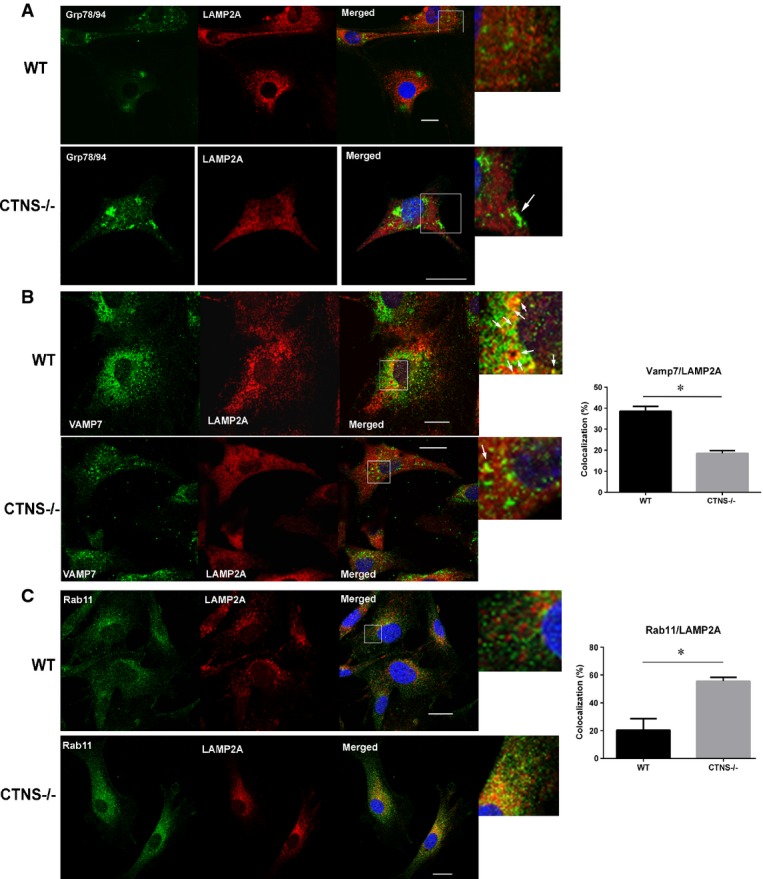
Abnormal localization of LAMP2A at trafficking vesicular compartments in CTNS-deficient cells Immunofluorescence analyses of endogenous LAMP2A, ER (Grp78/94) (A), which is expanded in cystinotic cells (white arrow), VAMP7-positive vesicles (B) and Rab11a-positive structures (C) was performed using wild-type or *Ctns*^*−*/*−*^ fibroblasts as described in Materials and Methods. At least 2 independent experiments were performed for each vesicular trafficking or ER marker. (B, C) Results are expressed as the percentage of LAMP2A that colocalizes with VAMP7 or Rab11. For Rab11/LAMP2A colocalization, 29 wild-type and 15 *Ctns*^*−*/*−*^ cells were quantified. For VAMP7/LAMP2A colocalization (white arrows), 49 WT and 57 *Ctns*^*−*/*−*^ cells were analyzed. Data are expressed as mean ± SEM. *P < 0.001 (unpaired *t*-test). Scale bars: 20 μm.

Further immunofluorescence analyses of endogenous LAMP2A in *Ctns*^*−*/*−*^ cells show that LAMP2A does not colocalize with ER markers ruling out entrapment of this protein in the ER (Fig6A). However, we found that LAMP2A, which colocalizes with VAMP7 on structures mostly distributed at the perinuclear area in wild-type cells, has significantly less colocalization with VAMP7 in *Ctns*^*−*/*−*^ cells (Fig6B). The staining of endogenous VAMP7 shown here is the expected for an endosomal protein and is similar to that shown by others (Fraldi *et al*, 2010). Since VAMP7 regulates late endosome–lysosome fusion (Ward *et al*, 2000) and mediates direct Golgi to lysosome translocation of lysosomal membrane-associated proteins (Pols *et al*, 2013), our data may suggest that trafficking of LAMP2A is altered in *Ctns*^*−*/*−*^ cells. Finally, we found that LAMP2A localizes to Rab11a-positive vesicles in *Ctns*^*−*/*−*^ cells but not in wild-type cells (Fig6C) further supporting possible alternative trafficking mechanisms for LAMP2A in cystinosis.

**Figure 7:**
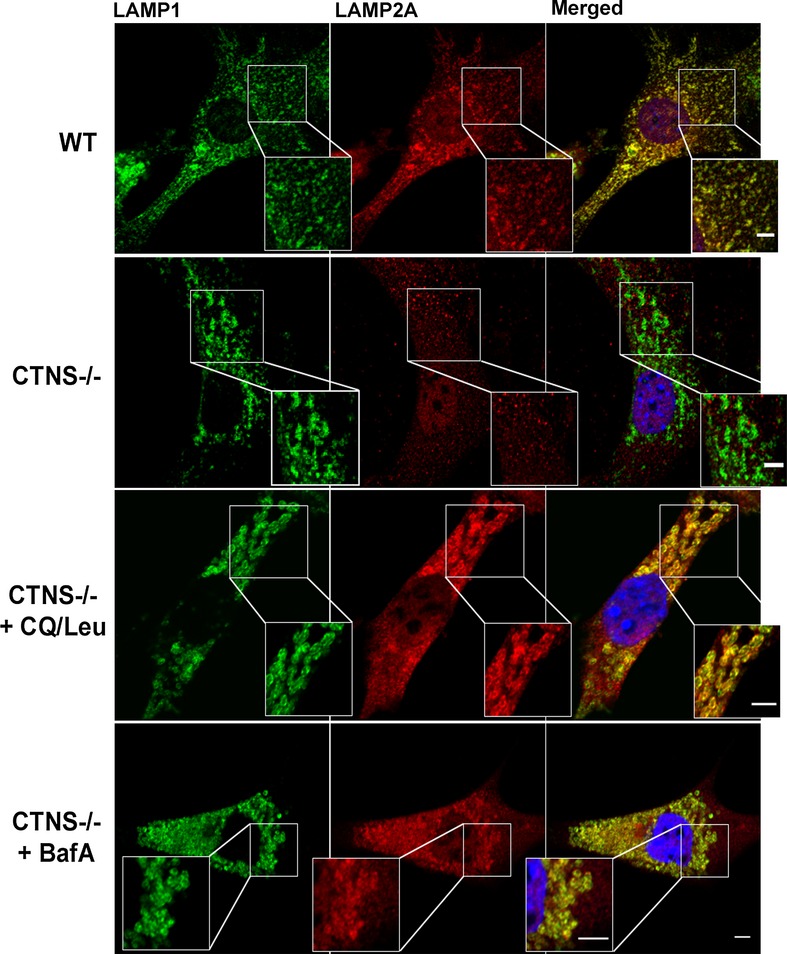
Lysosomal localization of LAMP2A in *Ctns*^*−*/*−*^ cells is rescued by inhibition of lysosomal proteases WT,*Ctns*^*−/−*^ or *Ctns*^*−/−*^ mouse fibroblasts treated for 20 h with either a combination of both leupeptin and chloroquine (CQ/Leu) or bafilomycin A (BafA) alone were fixed and immunostained with antibodies recognizing endogenous LAMP1 and LAMP2A proteins and samples were analyzed by confocal microscopy as described in Material and Methods. Lysosomal colocalization of LAMP1 and LAMP2A was evident in treated (middle lower and bottom panels) but not in untreated (middle upper panels) *Ctns*^*−*/*−*^ cells. Scale bars: 5 μm. Inset scale bars: 2 μm.

Post-transcriptionally, LAMP2A expression at the lysosomal membrane is regulated by targeted degradation by lysosomal proteases, upon its cleavage from the lysosomal membrane (Cuervo & Dice, 2000a; Bejarano & Cuervo, 2010). For this reason, to assess whether excessive degradation in the lysosome contributes to the decreased expression of LAMP2A in *Ctns*^*−/−*^ cells, *Ctns*^*−/−*^ fibroblasts were treated with various lysosomal inhibitors, namely the protease inhibitor leupeptin (Leu) in combination with chloroquine (CQ), or bafilomycin A (BafA) alone and LAMP2A was detected by immunofluorescence analysis. As shown in Fig7, treatment of *Ctns*^*−/−*^ fibroblasts with Leu/CQ or with BafA increased the detectable levels of LAMP2A at the lysosomal membrane as detected by its colocalization with the lysosomal marker LAMP1. Altogether, our data suggest that LAMP2A is characterized by a dual defective phenotype in *Ctns*^*−/−*^ cells that includes partial mislocalization of LAMP2A and increased lysosomal degradation of those LAMP2A molecules that are correctly targeted to the lysosome.

### Chaperone-mediated autophagy is impaired in cystinotic mice

**Figure 8:**
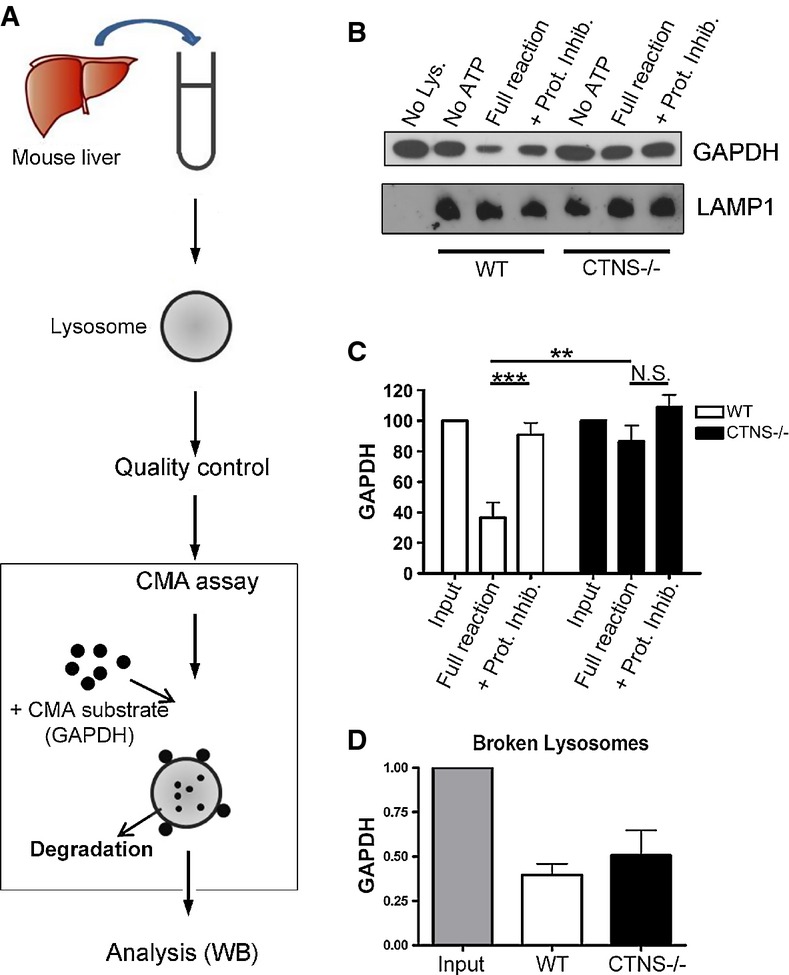
Chaperone-mediated autophagy is impaired in CTNS-deficient mice

LAMP2A-mediated substrate binding and internalization are essential processes for CMA and, in fact, changes in LAMP2A levels, or changes in its sub-compartmentalization to the lysosomal membrane, all contribute to modulate CMA activity (Cuervo & Dice, 2000a,b; Massey *et al*, 2006a; Kaushik & Cuervo, 2012). To test whether LAMP2A downregulation and mislocalization in CTNS-deficient cells resulted in impaired CMA activity, we validated and optimized an assay (Kaushik & Cuervo, 2008; Malkus & Ischiropoulos, 2012) to assess the ability of isolated CTNS-deficient lysosomes to digest CMA substrates (Fig8A). To avoid that increased lysosomal size and lysosomal overload in cystinotic cells (Johnson *et al*, 2013) could result in loss of the relevant lysosomal fraction in gradient techniques, we decided to use an established isolation method in which lysosomes are purified by sucrose sedimentation (Malkus & Ischiropoulos, 2012) (see Supplementary Materials and Methods). We optimized this assay to obtain CMA-active lysosomes, whose purity was verified by Western blot and integrity was verified by measuring β-hexosaminidase activity in purified lysosomes compared to burst lysosomes, as described in Supplementary Materials and Methods and shown in Supplementary Fig S4. Thus, lysosomes were free of mitochondria, ER and cytosolic contaminants (Supplementary Fig S4). Preparations containing >15% of broken lysosomes were discarded. For CMA assays, isolated lysosomes purified from livers of starved WT and *Ctns*^*−/−*^ mice were incubated with the CMA substrate, GAPDH, as represented in Fig8A and described in Materials and Methods. Using this procedure, we found that cystinotic lysosomes are characterized by defective degradation of the CMA substrate GAPDH (Fig8B and C), indicating that CMA is impaired in *Ctns*^*−/−*^ mice.

Schematic representation of lysosomal isolation and *in vitro*CMA assay [adapted from Kaushik and Cuervo (2008)].

Lysosomes were isolated from livers of starved wild-type (WT) and *Ctns*^*−/−*^ mice as described in Supplementary Materials and Methods and incubated at 37°C for 30 min with the CMA substrate GAPDH, in the presence or absence of ATP (necessary for CMA) and protease inhibitors (Prot. Inhib.). A fraction of the CMA reactions was then mixed with sample buffer and boiled at 95°C for 5 min, followed by SDS–PAGE and GAPDH and LAMP1 immunoblotting.

Quantitative densitometry analysis of CMA activity performed in independent experiments using lysosomes isolated from a total of 11 WT and 10 *Ctns*^*−/−*^ mice. Results are mean ± SEM. ***P = 0.0005; **P = 0.0041; N.S., not significant (unpaired *t*-test).

The ability of lysosomal proteases to mediate GAPDH degradation was assessed by incubating Triton X-100-treated WT and *Ctns*^*−/−*^ lysosomes with GAPDH for 30 min in acidic reaction buffer and compared with input (reaction with no lysosomes). Quantitative densitometry analysis of independent reactions performed with lysosomes from a total of 9 WT and 9 *Ctns*^*−/−*^ mice shows no significant difference in protease activity. Results are mean ± SEM. Source data are available online for this figure.

Substrate degradation by CMA is a multi-step process that involves substrate binding to the CMA receptor LAMP2A, substrate translocation into the lysosomal lumen and its subsequent degradation by lysosomal proteases (Bejarano & Cuervo, 2010; Kaushik & Cuervo, 2012). In order to define the mechanism of impaired substrate degradation by CMA in *Ctns*^*−/−*^ mice, we evaluated whether lysosomal proteases were functional in cystinotic mice. To this end, we incubated the CMA substrate GAPDH with Triton X-100-treated (broken) lysosomes, which showed similar rates of lysosomal degradation in preparations obtained from both WT and *Ctns*^*−/−*^ mice (Fig8D), indicating that impaired degradation of GAPDH by lysosomes from *Ctns*^*−/−*^ mice was not due to general impairment of lysosomal proteases.

### Cystinotic cells show increased susceptibility to oxidative stress-induced cell death

**Figure 9:**
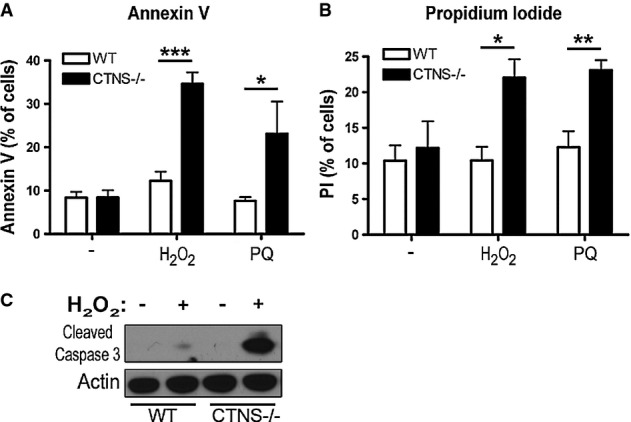
CTNS-deficient cells are more susceptible to oxidative stress-induced cell death

Activation of CMA is part of the cellular response to oxidative stress, and blockage of CMA in cultured cells decreases cellular viability following exposure to different pro-oxidant compounds (Kiffin *et al*, 2004; Massey *et al*, 2006a,b). Since CMA is impaired in cystinotic cells, we sought to verify whether this defect correlated with increased susceptibility to oxidative stress-induced cell death. To this end, WT and cystinotic cells were exposed to the pro-oxidants H_2_O_2_ and paraquat (PQ), which have previously been shown to induce excessive cell death in cells with defective CMA (Massey *et al*, 2006a), and cell viability was assessed by FACS analysis of annexin V and propidium iodide (PI) staining. As shown in Fig9A and B, annexin V and PI staining was increased in both H_2_O_2_- and PQ-treated *Ctns*^*−/−*^ cells compared to WT cells, indicating that cystinotic cells have enhanced susceptibility to oxidative stress-induced cell death. Furthermore, activation of caspase 3 was highly increased in *Ctns*^*−/−*^ mouse fibroblasts following mild exposure to H_2_O_2_ (Fig9C), further confirming increased susceptibility to oxidative stress-induced apoptotic cell death in these cells.

WT and *Ctns*^*−/−*^ mouse fibroblasts were treated with either 50 μM H_2_O_2_ or 1 mM paraquat (PQ) for 4 h and stained with (A) FITC-annexin V or (B) propidium iodide (PI) and analyzed by FACS. Results from 3 different experiments are shown as mean ± SEM. In (A), *P = 0.05; ***P = 0.0002. In (B), *P = 0.046; **P = 0.0065. Unpaired *t*-test.

WT and *Ctns*^*−/−*^ cells were treated as described above and samples analyzed by Western blot using antibodies recognizing cleaved (active) caspase 3. Data are representative of 2 independent experiments with similar results.

### CTNS expression but not reduction of lysosomal overload rescues LAMP2A mislocalization in *Ctns*^*−/−*^ cells

**Figure 10:**
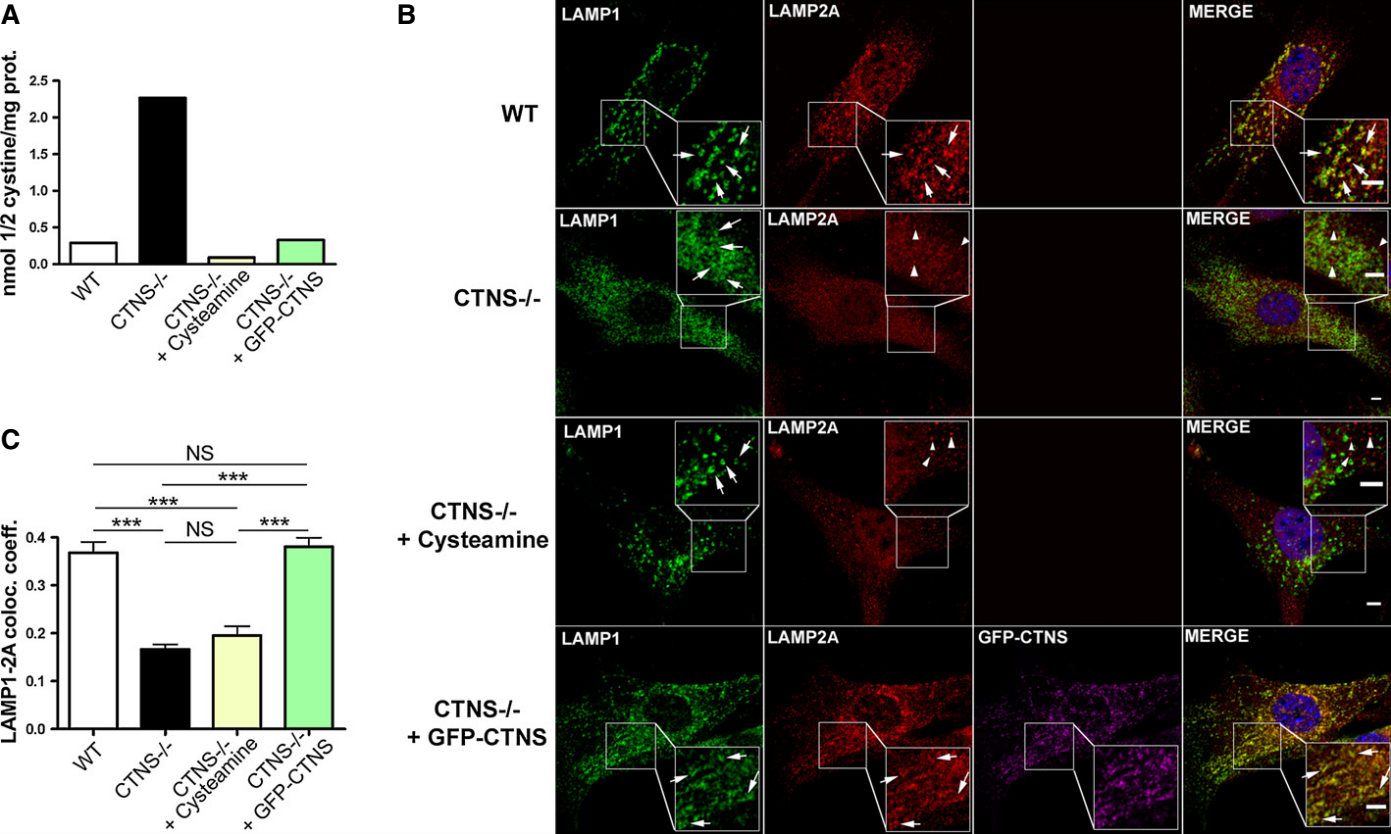
LAMP2A mislocalization is independent of cystine-induced lysosomal overload

Although cystine accumulation is toxic for cells, tissue injury in cystinotic patients occurs despite effective cystine depletion therapy (Cherqui, 2012). Therefore, it is important to determine whether the defective molecular mechanisms that contribute to cell dysfunction and tissue injury in cystinosis are dependent on lysosomal overload. For this reason, to assess whether defective CMA in cystinosis was due to cystine accumulation and lysosomal overload, we treated *Ctns*^*−/−*^ fibroblasts with cysteamine, which depletes lysosomal cystine accumulation (Butler & Zatz, 1984) and evaluated lysoso-mal localization of LAMP2A in these cells. Although cysteamine treatment depleted cystine accumulation and reduced lysosomal overload in *Ctns*^*−/−*^ cells (Fig10A), LAMP2A localization maintained a reticular distribution similar to that observed in untreated *Ctns*^*−/−*^ cells (Fig10B, red panels), and colocalization of LAMP2A with the lysosomal marker LAMP1 was not restored in cysteamine-treated cells (Fig10B (merge) and C). By contrast, lentiviral transduction of GFP-CTNS in CTNS-deficient fibroblasts, which also reduced intracellular cystine levels (Fig10A), was able to restore lysosomal localization of LAMP2A (Fig10B and C), indicating that lysosomal overload is not the primary cause of LAMP2A mislocalization in cystinotic cells and that CTNS deficiency itself induces improper LAMP2A localization.

Cystine content in WT,*Ctns*^*−/−*^, cysteamine-treated *Ctns*^*−/−*^ fibroblasts or *Ctns*^*−/−*^ fibroblasts expressing GFP-CTNS was assessed by mass spectrometry.

WT,*Ctns*^*−/−*^, *Ctns*^*−/−*^ fibroblasts treated with 1 mM cysteamine (48 h), or *Ctns*^*−/−*^ fibroblasts expressing GFP-CTNS were fixed and stained with anti-LAMP1 and LAMP2A antibodies as indicated. For better visualization of LAMP1/LAMP2A colocalization, GFP-CTNS was pseudocolored as magenta and LAMP1 was pseudocolored as green (lower panels). Some lysosomes are indicated with arrows. Arrowheads indicate LAMP2A distribution to structures different from lysosomes (only observed in *Ctns*^*−/−*^ cells and in cysteamine-treated *Ctns*^*−/−*^ cells). Rescue of the LAMP2A localization to lysosomes was observed in *Ctns*^*−/−*^ cells expressing GFP-CTNS. Scale bar: 2 μm.

Quantification of the colocalization analysis described in (B). Calculation of the Pearson's colocalization coefficient was done by analyzing 113 WT, 251 *Ctns*^*−/−*^, 88 cysteamine-treated and 164 GFP-CTNS-expressing *Ctns*^*−/−*^ cells, by using the ZEN 2010 software. Results are mean ± SEM. ***P < 0.001; NS, not significant (one-way ANOVA, Bonferroni's multiple comparisons test).

### Impaired CMA activity in CTNS deficiency despite effective *in vivo* reduction of lysosomal overload

**Figure 11:**
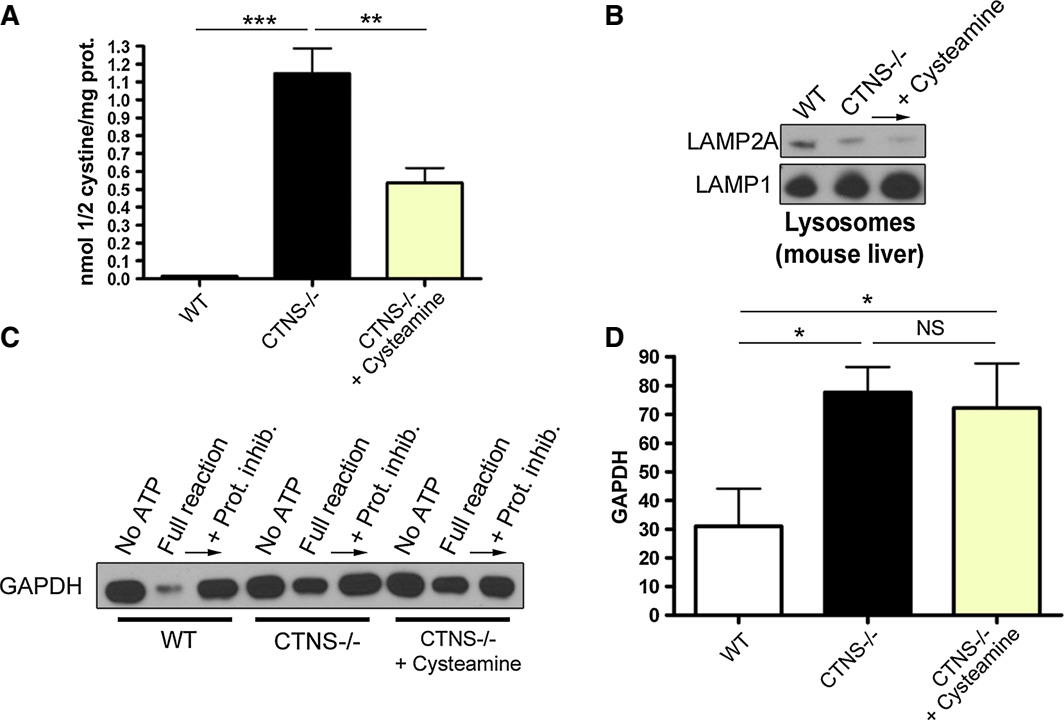
Cysteamine treatment does not rescue CMA impairment in *Ctns*^*−*/*−*^ mice

To determine whether reduction of lysosomal overload could rescue CMA defects *in vivo*, CTNS-deficient mice were treated for 5 days with cysteamine, starved, and the ability of wild-type and cystinotic lysosomes to mediate degradation of CMA substrates was assessed upon lysosomal isolation from mouse livers as described in Fig8A. Cysteamine treatment, despite significantly reducing intralysosomal cystine accumulation in CTNS-deficient mice (Fig11A) to levels similar to those observed in treated cystinotic patients (Belldina *et al*, 2003), did not rescue either reduced expression of LAMP2A on cystinotic lysosomes (Fig11B) or the CMA impairment in these mice (Fig11C and D). Altogether, our data support the idea that CMA impairment in cystinosis is not caused by lysosomal overload and suggest that CTNS deficiency is the main cause of CMA impairment in this disorder.

Cystine content in WT,*Ctns*^*−/−*^ and cysteamine-treated *Ctns*^*−/−*^ mouse livers was assessed by mass spectrometry. Results are mean ± SEM (*n *= 4). ***P < 0.001; **P = 0.0026 (one-way ANOVA, Bonferroni's multiple comparisons test).

Western blot analysis of LAMP2A expression in lysosomes isolated from WT,*Ctns*^*−/−*^ and cysteamine-treated *Ctns*^*−/−*^ mouse livers.

Lysosomes, isolated as in (B), were incubated with the CMA substrate GAPDH, and CMA activity was evaluated as in Fig8 and as described in Materials and Methods.

Quantitative analysis of substrate degradation is expressed as percent of the residual amount of GAPDH in the full reaction relative to its amount in the presence of protease inhibitors. Results are mean ± SEM (*n *= 4). *P = 0.0126 for WT versus *Ctns*^*−*/*−*^ and *P = 0.0436 for WT versus *Ctns*^*−*/*−*^ + cysteamine; NS, not significant; unpaired *t*-test. Source data are available online for this figure.

## Discussion

Cystine accumulation is the hallmark of cystinosis and is regarded as the primary defect of cell malfunction; however, renal injury is not seen in all human forms of cystinosis (Anikster *et al*, 1999), and progressive renal injury occurs despite cystine depletion therapy, indicating that other factors can contribute to disease progression independently of cystine accumulation. In the present work, we identified a novel mechanism of impaired CMA that is associated with CTNS deficiency but is independent of lysosomal overload. Using cellular and *in vivo* approaches, we found that the CMA receptor LAMP2A is downregulated and mislocalized in cystinotic cells and that lysosomal degradation of the CMA substrate GAPDH is impaired in lysosomes from *Ctns*^*−/−*^ mice. We also show that CMA defects are independent of lysosomal overload, suggesting that the absence of CTNS expression causes the defective autophagic balance in cystinosis. Since CMA activation is a major response mechanism to oxidative stress, which contributes to organ dysfunction in cystinotic patients (Vaisbich *et al*, 2011; Pache de Faria Guimaraes *et al*, 2014), our findings suggest that CMA impairment in cystinosis represents a significant contributor to tissue damage in the pathogenesis of the disease.

LAMP2A is the main regulator of CMA and its expression level at the lysosomal membrane directly correlates with CMA activity (Cuervo & Dice, 2000a). Here, we show that cystinosis is characterized by impaired CMA associated with a marked decrease of LAMP2A expression in cells and tissues and with reduced localization of LAMP2A at the lysosomal membrane (Figs4 and 5). Our finding showing that inhibition of lysosomal proteases increases the level of LAMP2A detected at the lysosomal membrane in CTNS-deficient cells (Fig7) suggests that reduced LAMP2A at the lysosomal membrane is in part explained by excessive lysosomal degradation. This is in agreement with previous studies showing that LAMP2A levels are regulated by degradation by lysosomal proteases (Kaushik & Cuervo, 2012). The phenotype observed in cystinotic cells is specific for LAMP2A since the other major LAMP2 isoform present in fibroblasts, LAMP2B, is normally expressed and localized at lysosomes in *Ctns*^*−*/*−*^ cells. Previous observations that LAMP2A stabilization at the lysosomal membrane is regulated by glial fibrillary acidic protein which binds to LAMP2A through its unique carboxy-terminal domain at the cytosolic tail (Kaushik & Cuervo, 2012) support that LAMP2A and LAMP2B turnover have independent regulation. Furthermore, differential turnover of LAMP2 proteins have been directly demonstrated in previous studies showing that the half-life of LAMP2A at the lysosomal membrane is significantly shorter than that of other LAMP2 proteins in fed conditions (Fig 1C in Cuervo & Dice, 2000a). Our results support those findings and highlight their relevance in cystinosis.

LAMP2A degradation has been suggested to take place at specific lipid microdomains of the lysosomal membrane (Kaushik *et al*, 2006; Bandyopadhyay *et al*, 2008; Rodriguez-Navarro *et al*, 2012), where LAMP2A is retained as a monomer and is targeted for proteolytic degradation (Cuervo *et al*, 2003; Kaushik *et al*, 2006). A possible explanation for the increased degradation of LAMP2A in cystinosis is that the absence of CTNS could induce changes in the fluidity of the lysosomal membrane, resulting in subsequent sequestration of LAMP2A into sub-domains where its proteolysis is exacerbated. Our finding that CTNS expression, but not reduction of lysosomal overload, increases the detectable levels of LAMP2A at the lysosomal membrane (Fig10) supports the idea that CTNS may help stabilize LAMP2A at the lysosomal membrane either directly or indirectly.

In addition to the increased susceptibility to degradation at the lysosomal membrane, our data showing partial localization of LAMP2A at non-lysosomal structures suggest that LAMP2A trafficking is also impaired in *Ctns*^*−*/*−*^ cells (Fig5). The trafficking of lysosomal-associated membrane proteins to the lysosome invol-ves several mechanisms including a direct pathway through the endolysosomal system and an indirect pathway that involves trafficking to the plasma membrane and subsequent re-routing to the endosomes (Saftig & Klumperman, 2009). Another mechanism consisting of clathrin-independent VAMP7/Vps41-mediated transport of LAMP carrier vesicles from the TGN to late endosomes was recently described (Pols *et al*, 2013). The poor colocalization of LAMP2A with the SNARE VAMP7 in *Ctns*^*−*/*−*^ cells compared to wild-type cells (Fig6B) raises the question of whether the VAMP7-dependent transport of LAMP2A is defective in cystinosis, and suggests that further examination of the VAMP7-dependent transport system in cystinosis would be necessary to further elucidate this question. Instead, LAMP2A was found in Rab11-positive trafficking vesicles in cystinosis suggesting that alternative mechanisms of transport may be functional. Thus, the partial localization of LAMP2A at non-lysosomal structures and its increased degradation are not necessarily mutually exclusive phenotypes since post-translational modification may affect different pools of LAMP2A in dissimilar ways. Supporting this, it was proposed that post-translational modifications can fine-tune both the sorting signals and stability of lysosomal membrane proteins (Barriocanal *et al*, 1986; Saftig & Klumperman, 2009). Defects in ER initiated post-translational modifications in cystinosis are suggested by previous studies, showing high levels of ER stress and expansion in cystinotic cells (Johnson *et al*, 2013). The trafficking mechanisms and posttranslational modifications regulating LAMP2A transport in cystinosis are currently under investigation in our laboratory.

Differently from other LSDs (Settembre *et al*, 2008a,b), we show that macroautophagy is not impaired in cystinotic cells, as autophagosome maturation, digestion of the autophagic content, long-lived protein degradation and p62 degradation rates were found to be functional in *Ctns*^*−/−*^ cells. However, we found that cystinotic cells and tissues have an elevated number of autophagosomes. The regulation of different autophagic pathways is closely interconnected and most cells activate macroautophagy in response to blockage of CMA (Massey *et al*, 2006a; Kaushik *et al*, 2008). Thus, it is possible that the increased number of autophagosomes in cystinotic cells is a result of compensatory activation due to CMA impairment. This is supported by the evidence that reconstitution of *Ctns*^*−/−*^ cells with GFP-CTNS expression led to increased distribution of LAMP2A at lysosomal membranes, which correlated with normalization of LC3B-II levels despite the fact that the activity of mTOR was not affected.

Despite their compensatory effect, macroautophagy and CMA are not redundant. For instance, previous studies performed in cells with compromised CMA suggested that compensatory activation of macroautophagy is not always sufficient to ensure cell survival upon exposure to stressors (Massey *et al*, 2006a). In particular, activation of CMA is part of the cellular response to oxidative stress (Kiffin *et al*, 2004; Bejarano & Cuervo, 2010), and blockage of CMA in cultu-red cells decreases cell viability following exposure to different pro-oxidant compounds (Kiffin *et al*, 2004; Massey *et al*, 2006a,b), a process that is not prevented even in conditions of increased macroautophagy. In this work, we found that cystinotic cells show an increased susceptibility to oxidative stress-induced cell death. Increased oxidative stress has also been found in cystinotic patients (Vaisbich *et al*, 2011). Remarkably, a recent clinical trial showed significant improvement of renal function in cystinotic patients treated with the anti-oxidant drug N-acetylcysteine (NAC) (Pache de Faria Guimaraes *et al*, 2014), indicating that oxidative stress can contribute to the progression of renal disease in cystinosis patients even when they show a significant response to treatment with cysteamine (Gahl *et al*, 2007; Vaisbich & Koch, 2010; Vaisbich *et al*, 2011). In this context, our findings that LAMP2A mislocalization and CMA impairment are not dependent on lysosomal overload are highly relevant and represent a direct evidence of an impaired cellular mechanism not linked to substrate storage in cystinosis. Thus, our findings not only highlight that CMA impairment may represent an important contributor to oxidative stress-induced tissue damage in the pathogenesis of the disease, but they can also help explain why cysteamine treatment is not fully effective in cystinosis treatment.

In conclusion, we found that CMA but not macroautophagy is impaired in cystinosis. Due to the importance of CMA in cell homeostasis and response to stress, we suggest that CMA impairment represents an important contributor to disease progression in this disorder. Our findings highlight that lysosomal overload is not the only mediator of cell malfunction in cystinosis and identifies correction of CMA as a potential additional strategy for the treatment of cystinosis.

## Materials and Methods

### Animal models

All animal studies were performed in compliance with the United States Department of Health and Human Services Guide for the Care and Use of Laboratory Animals. All studies were conducted according to National Institutes of Health and institutional guidelines and with approval of the animal review boards at The Scripps Research Institute and UCSD.

The C57BL/6 *Ctns*^−/−^ mice were described before (Nevo *et al*, 2010). For analysis of autophagosomes *in vivo*, C57BL/6 GFP-LC3^+/+^ transgenic mice (Mizushima *et al*, 2004) were crossed with C57BL/6 *Ctns*^*−/−*^ mice (Nevo *et al*, 2010) to generate *Ctns*^*−/−*^ mice expressing GFP-LC3 (C57BL/6 *Ctns*^*−/−*^ GFP-LC3^+/+^). Mice were genotyped as described (Mizushima *et al*, 2004; Nevo *et al*, 2010). The GFP-LC3^+/+^ mice were provided by the RIKEN BRC through the National Bio-Resource Project of the MEXT, Japan. All mouse studies were carried out together with age- and sex-matched controls. For the analysis of LC3 expression in mouse tissues, 3 C57BL/6 GFP-LC3^+/+^ transgenic mice and 3 C57BL/6 *Ctns*^*−/−*^ GFP-LC3^+/+^ were used. For CMA activity and LAMP2A expression, a total of 19 C57BL/6 wild-type and 24 C57BL/6 *Ctns*^−/−^ mice were utilized. All mice used were between 8 and 12 weeks old.

Neonatal skin fibroblasts from *Ctns*^−/−^ and wild-type mice were prepared by standard procedures (Johnson *et al*, 2013). Neonatal murine fibroblasts and 293T cells (ATCC) were maintained in Dulbecco's modified Eagle's medium (Gibco) supplemented with 10% FBS (Corning Cellgro) and penicillin/streptomycin/glutamine (Life Sciences). Bone marrow was collected from femurs and tibia of mice and was used to prepare BMDMs, which were cultured in DMEM supplemented with 10% FBS and 20% L929-conditioned medium in the presence of antibiotics (Life Sciences).

### Starvation and recovery protocols

For macroautophagy studies, two starvation protocols were used: Protocol 1 consisted of complete nutrient deprivation (amino acid/serum starvation), in which WT and *Ctns*^*−/−*^ cells were washed briefly in HBSS (Gibco) and media-aspirated, and fresh HBSS was added again, followed by 45 min of incubation at 37°C, in the presence or absence of the lysosomal inhibitors bafilomycin A (LC laboratories, 100 nM), leupeptin (Sigma, 20 μM) or chloroquine (Sigma, 50 μM). Protocol 2 was serum starvation only: cells were briefly washed in serum-free DMEM (containing 1× amino acids) and media-aspirated, and fresh serum-free DMEM was added followed by 5 h incubation at 37°C, in the presence or absence of lysosomal inhibitors. For mTOR activity experiments, cells were starved using either protocol 1 or 2, and recovery was achieved by replacing starvation media with DMEM supplemented with 10% FBS and antibiotics, followed by 1 h incubation at 37°C. For CMA studies, starvation was induced as in protocol 2, followed by 20–24 h incubation at 37°C.

### Cysteamine treatment of cultured cells and animals and cystine quantification

WT and *Ctns*^*−/−*^ cells were treated for 48 h with 1 mM cysteamine in normal growth medium, and medium containing fresh cysteamine was replaced every 12 h. For *in vivo* studies, *Ctns*^*−/−*^ mice were intraperitoneally injected with 30 mg/kg cysteamine every 12 h for 5 days. For cystine quantification in cells and tissues, cystine was extracted as described (Johnson *et al*, 2013) and cystine levels were measured by mass spectrometry at the Biochemical Genetics Laboratory, University of California, San Diego.

### CMA assay

For CMA assays, 25 μg of purified lysosomes were incubated for 30 min at 37°C with 2 μg of purified recombinant GAPDH (Sigma), 10 μl of “6× energy regenerating system” (60 mM MgCl_2_, 60 mM ATP, 12 mM phosphocreatine, 30 μg creatine phosphokinase, in 0.25 M sucrose, pH 7.4), 0.6 μg Hsc70 (Enzo Life Science) and brought up to 60 μl with reaction buffer (10 mM MOPS, pH 7.3, 0.25 M sucrose, 5.4 μM cysteine, 1 mM DTT). Control conditions were run in parallel with either no lysosomes, no energy regenerating system or by addition of EDTA-protease inhibitors cocktail (Roche). After incubation at 37°C for 30 min, a fraction of the CMA reactions was mixed with sample buffer and boiled at 95°C for 5 min, followed by SDS-PAGE and GAPDH immunoblotting.

### Degradation of long-lived proteins

Degradation of long-lived proteins was measured using radioactive ^35^S-methionine as described in Supplementary Materials and Methods.

### Immunoblotting, immunofluorescence, confocal microscopy and high-resolution microscopy

Immunoblotting, immunofluorescence analyses and microscopy approaches are described in Supplementary Materials and Methods.

### Lysosome fractionation and β-hexosaminidase assay

The purification of the lysosomal fractions for CMA assays was performed by a modified protocol based on methods previously described (Kaushik & Cuervo, 2008; Malkus & Ischiropoulos, 2012). Details are given in Supplementary Materials and Methods.

### Analysis of cell death and flow cytometry assays

Cell death was measured by flow cytometry using FITC-annexin V and propidium iodide (PI) staining (BD Pharmingen), as described in Supplementary Materials and Methods.

### Statistical analysis

Data are presented as means, and error bars correspond to standard errors of the means (SEMs), unless otherwise indicated. Statistical significance was determined using the unpaired Student's *t*-test or the one-way ANOVA, Bonferroni's multiple comparisons test using GraphPad InStat (version 3) or GraphPad Prism (version 6) softwares, and graphs were made using GraphPad Prism (version 6) software. Exact *P*-values are notified unless *P *< 0.001.

## The paper explained

### Problem

Lysosomal storage disorders (LSDs) are multi-systemic diseases characterized by lysosomal overload and cellular dysfunction. Although substrate reduction and lysosomal overload-decreasing therapies may delay disease progression, tissue injury occurs despite depletion therapies. Thus, in order to improve treatment of LSDs, it is crucial to understand the defective molecular mechanisms that could lead to cell dysfunction and tissue injury in addition to the defects induced by lysosomal overload.

### Results

In the present work, we study autophagic pathways in cystinosis, a LSD caused by defects in the cystine transporter cystinosin (CTNS). We show that cystinotic cells and tissues are characterized by an increase in autophagosome number. However, different from other LSDs, macroautophagy flux is not impaired in cystinosis while mTOR activity was not affected. Conversely, we identify a mechanism of impaired CMA, a selective form of autophagy with essential roles in cell homeostasis and stress response. We show that cystinotic cells are characterized by aberrant expression and localization of the CMA receptor LAMP2A, which results in defective lysosomal degradation of CMA substrates in cystinotic mice. Importantly, LAMP2A and CMA defects were independent of lysosomal overload but associated with CTNS deficiency itself.

### Impact

Due to the role of CMA as a major response pathway to oxidative stress, which is an established mediator of tissue damage in cystinosis, our results highlight CMA impairment as an important pathogenic factor in CTNS deficiency. Our data also indicate that lysosomal overload is not the only mediator of cell dysfunction in LSDs, underlin-ing the need for treatments complementary to substrate depletion therapies.

## Supporting Information

Supplementary Information

Source Data for Supplementary Figure S1A

Review Process File

Source Data for Figure 2 C D E

Source Data for Figure 3A

Source Data for Figure 4D

Source Data for Figure 8B

Source Data for Figure 11C
