# Supplementary figures and images for: Impairment of chaperone-mediated autophagy leads to selective lysosomal degradation defects in the lysosomal storage disease cystinosis
Source: EMBO Mol Med. 2015 Jan 13;7(2):158–74. doi: 10.15252/emmm.201404223 (PMC4328646; doi:10.15252/emmm.201404223)

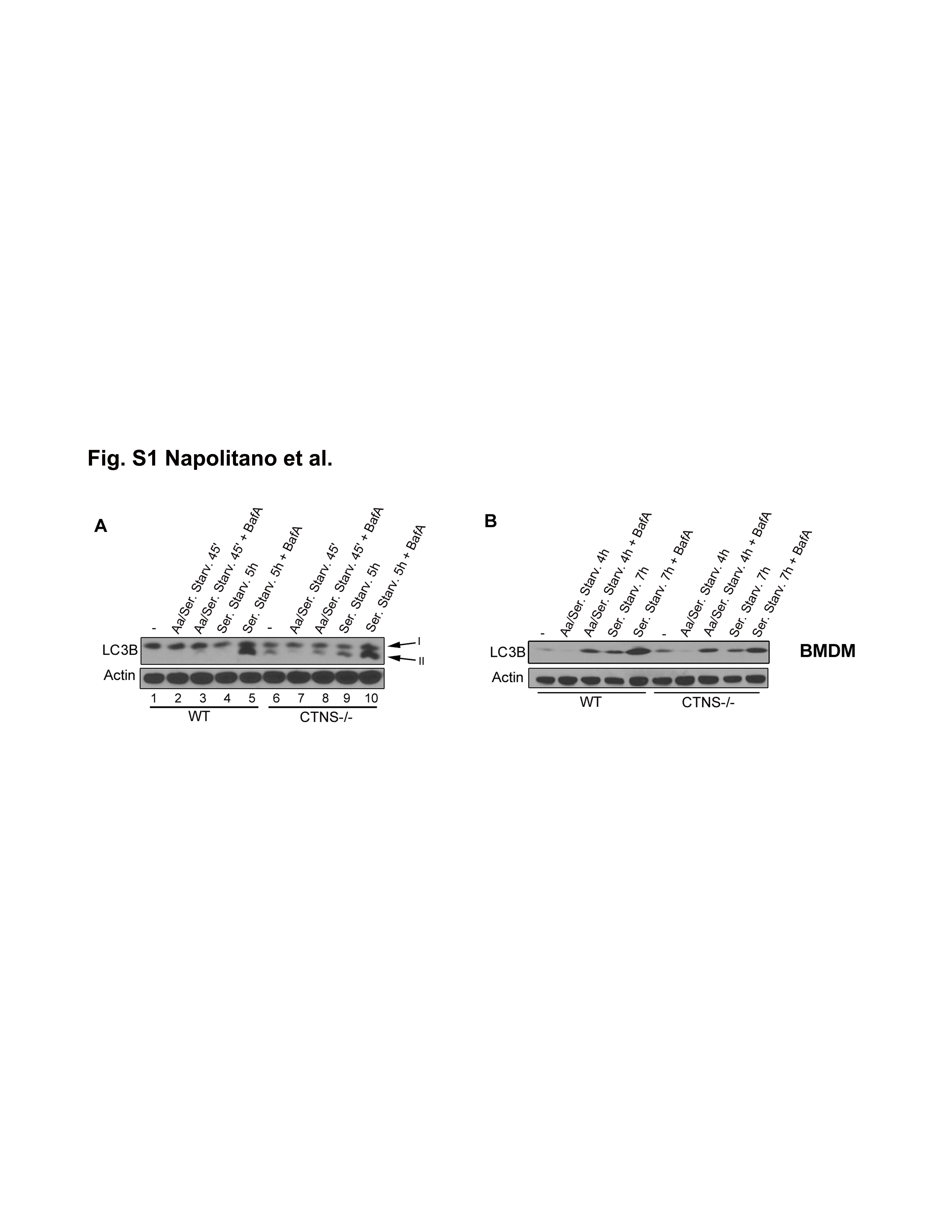

Supplement: Supplementary file 1 [file emmm0007-0158-sd1.tif]

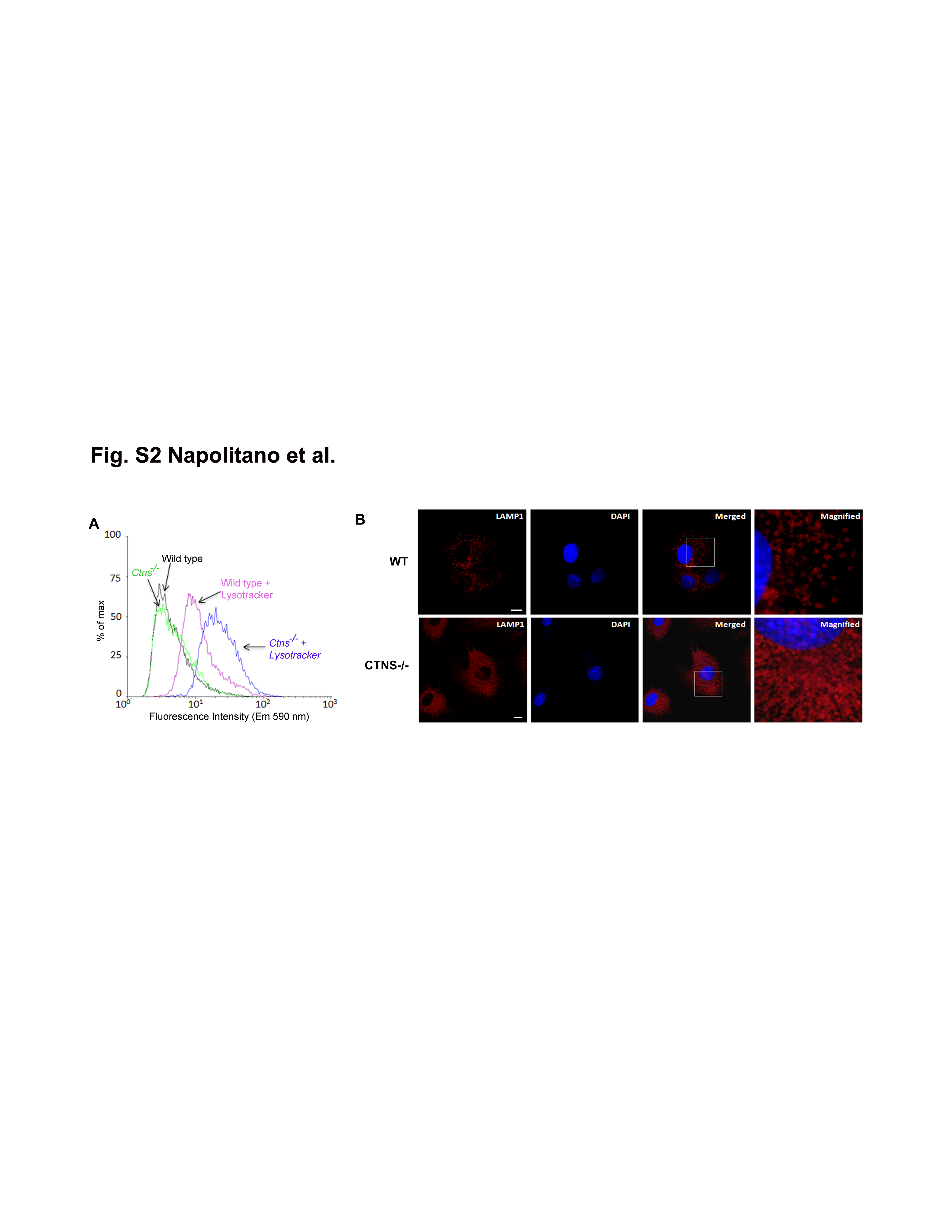

Supplement: Supplementary file 2 [file emmm0007-0158-sd2.tif]

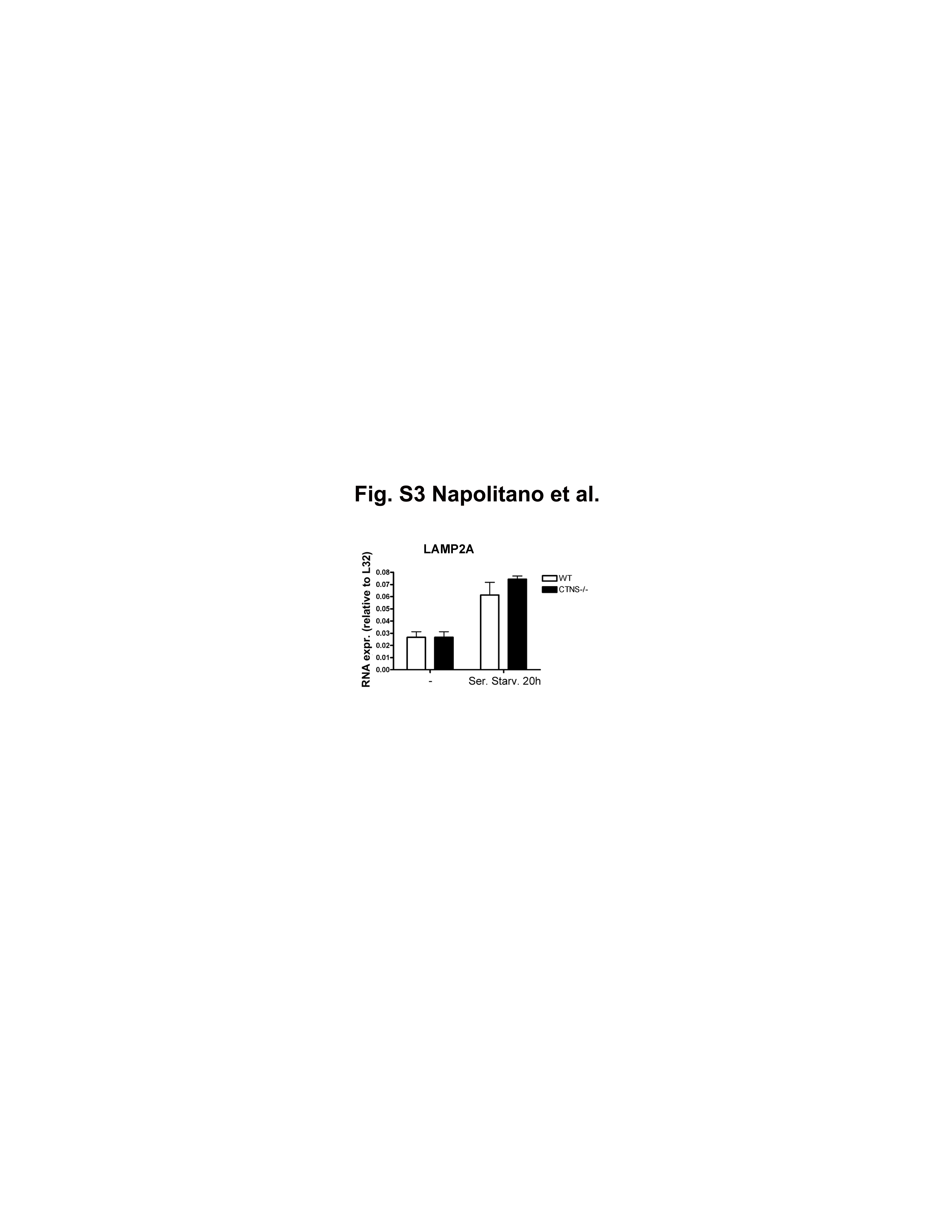

Supplement: Supplementary file 3 [file emmm0007-0158-sd3.tif]

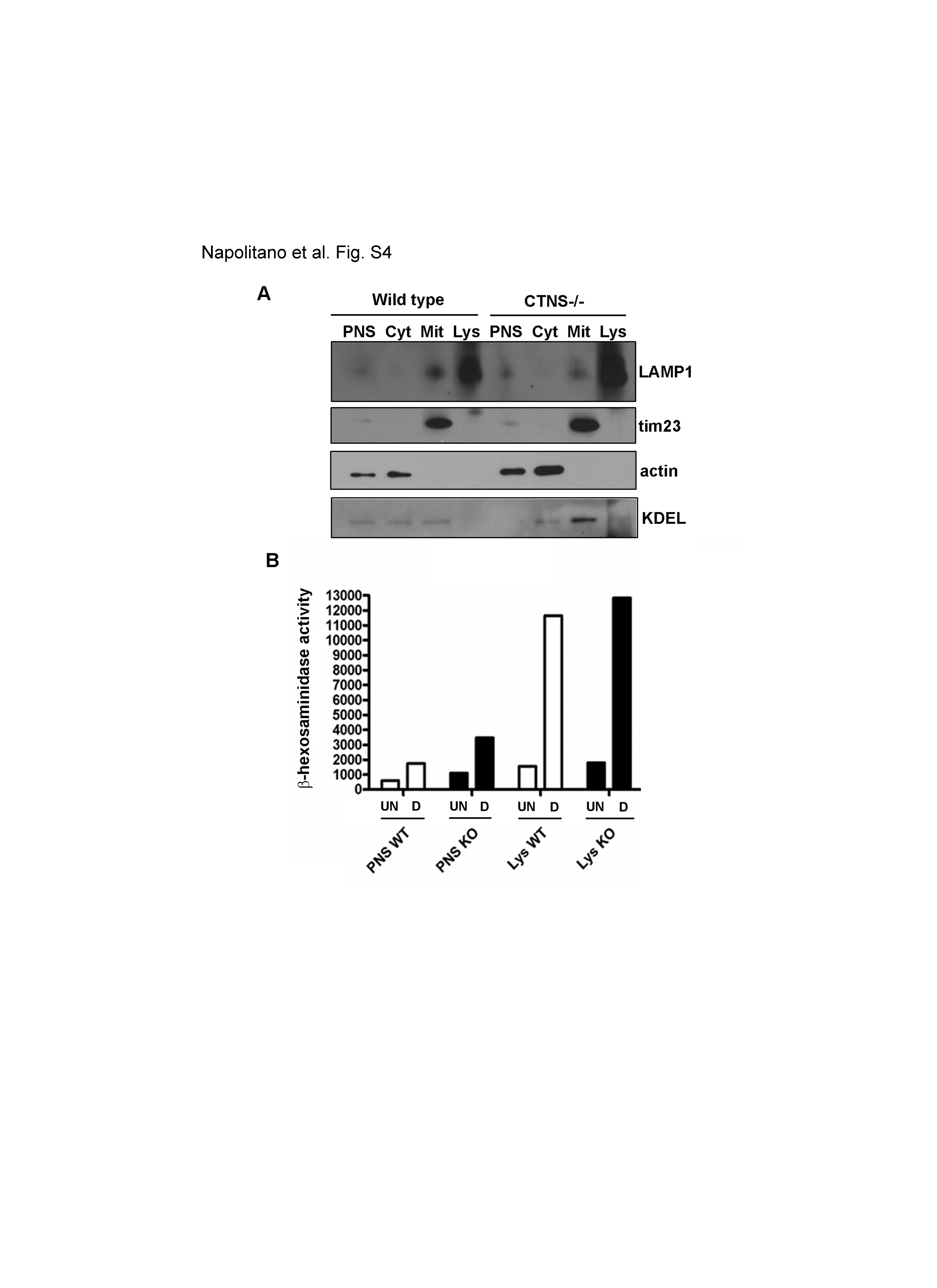

Supplement: Supplementary file 4 [file emmm0007-0158-sd4.tif]

Figure S1A

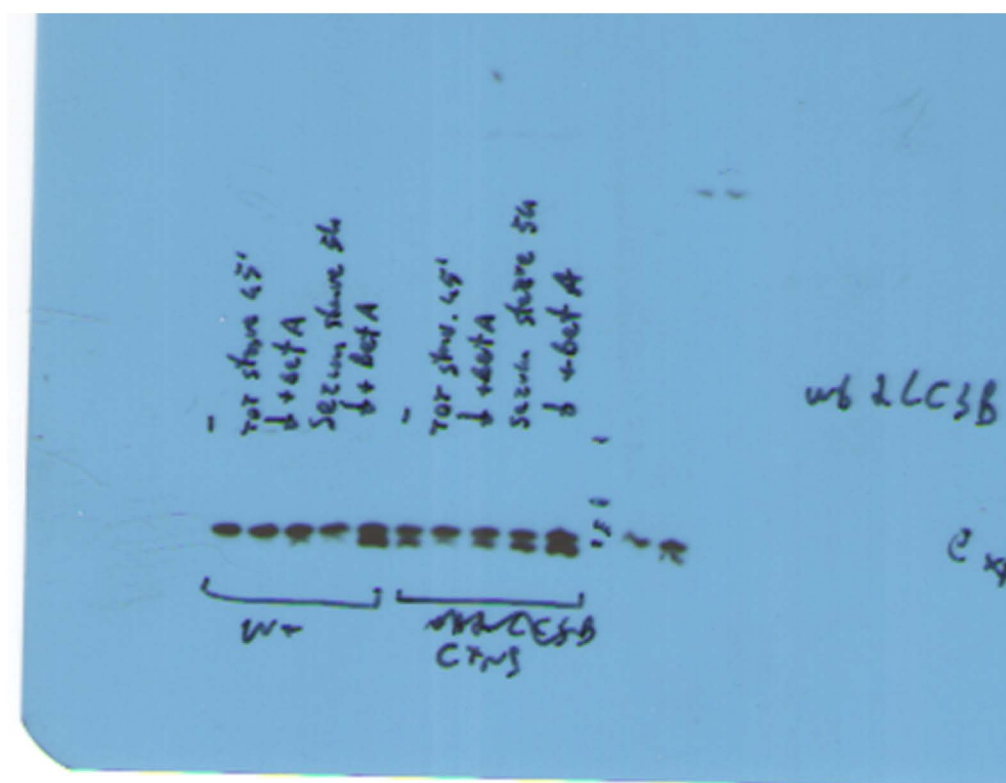

Supplement: Supplementary file 6 [file emmm0007-0158-sd6.pdf]

Figure 2C

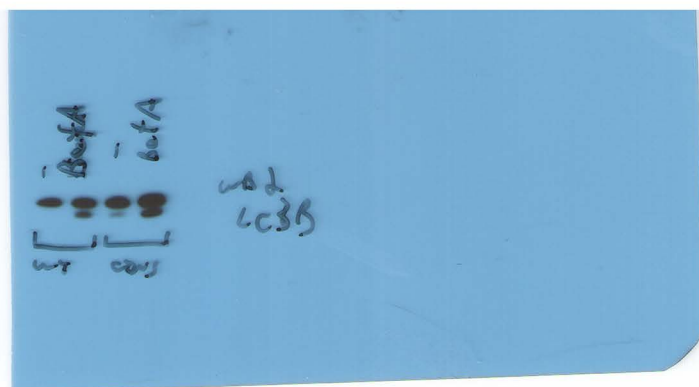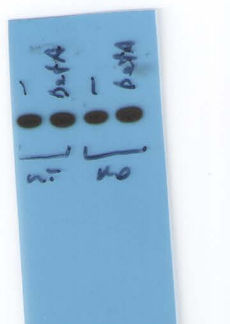

Figure 2D

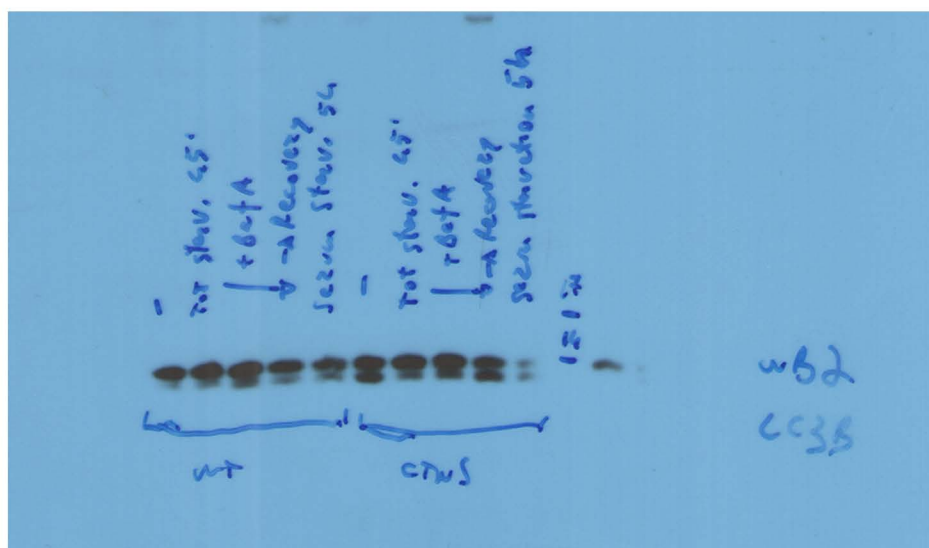

Figure 2E

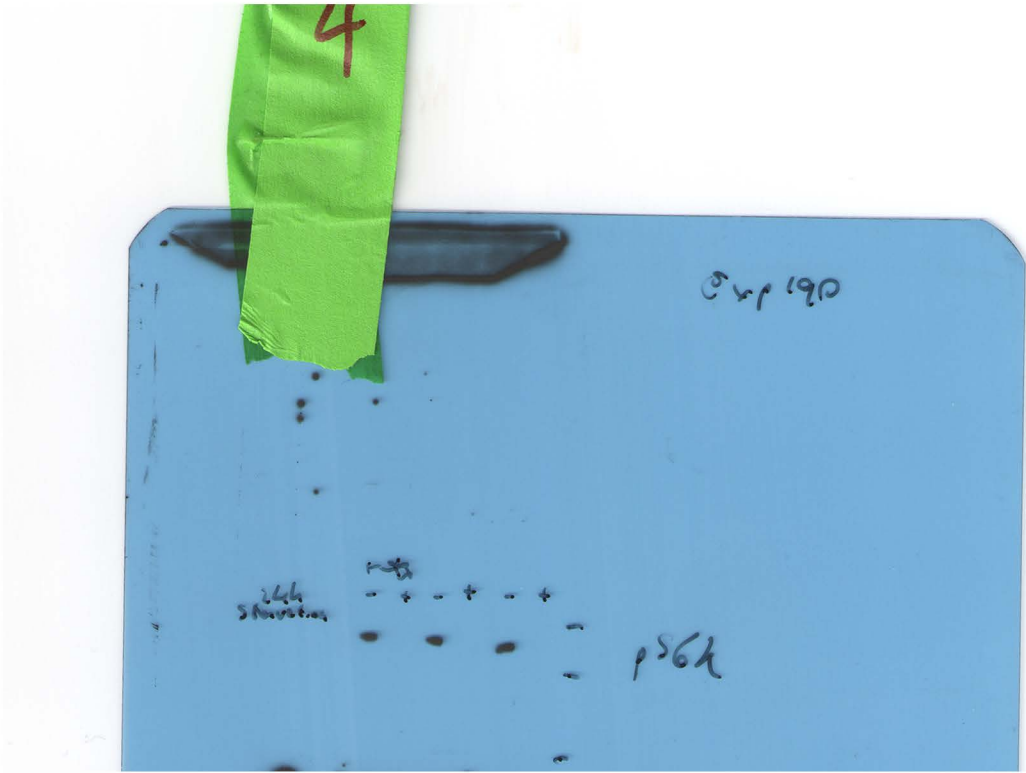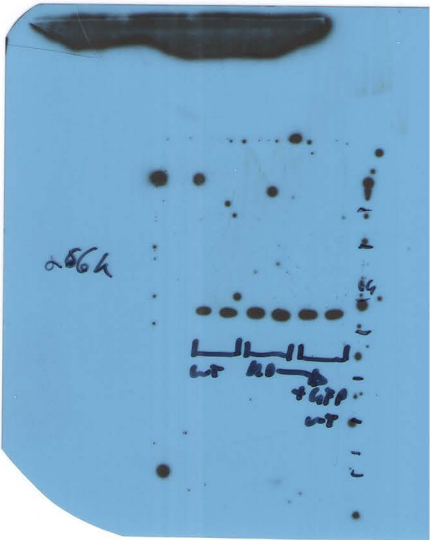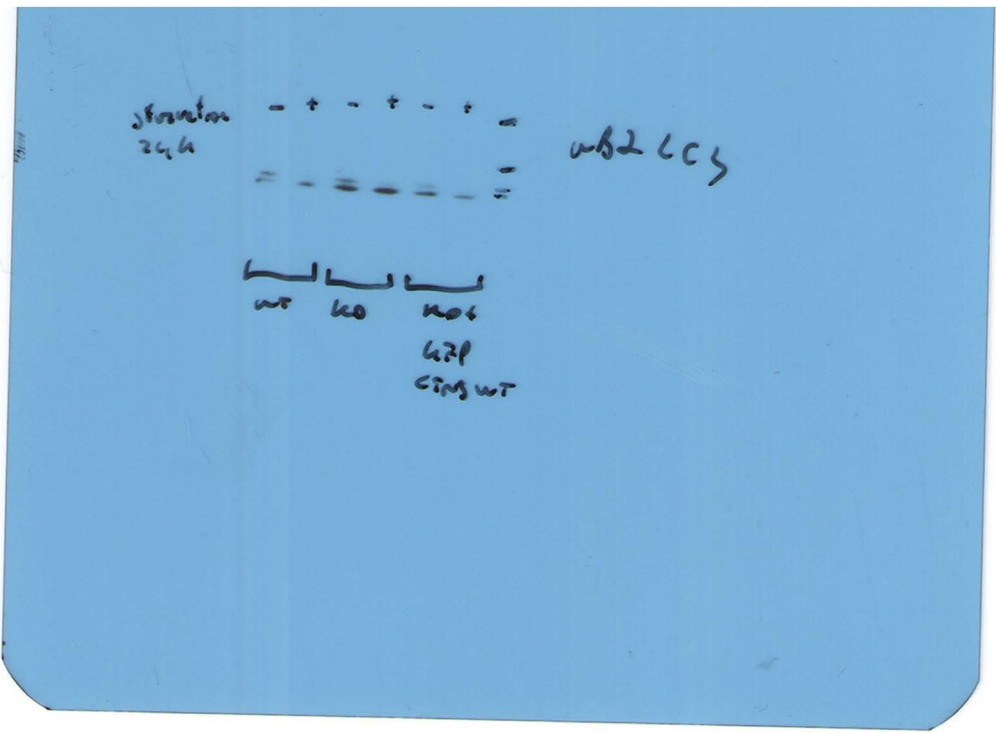

Supplement: Supplementary file 8 [file emmm0007-0158-sd8.pdf]

Figure 3A

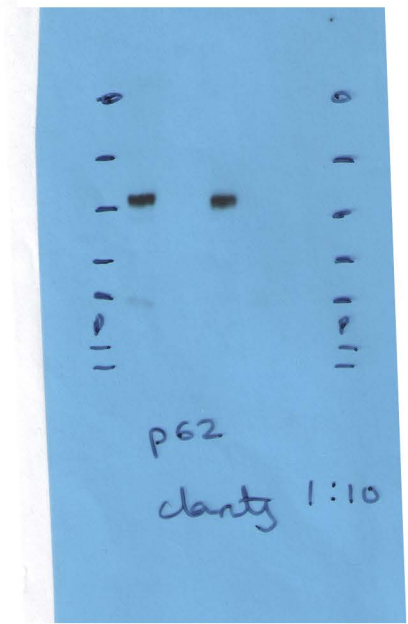

Supplement: Supplementary file 9 [file emmm0007-0158-sd9.pdf]

Figure 4D

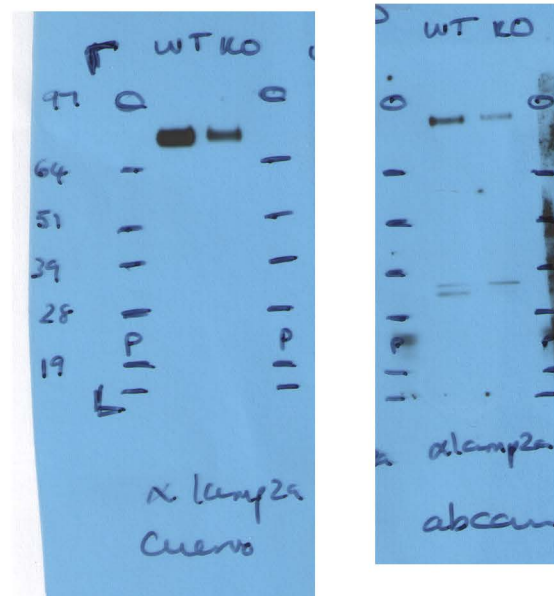

Supplement: Supplementary file 10 [file emmm0007-0158-sd10.pdf]

Figure 8B

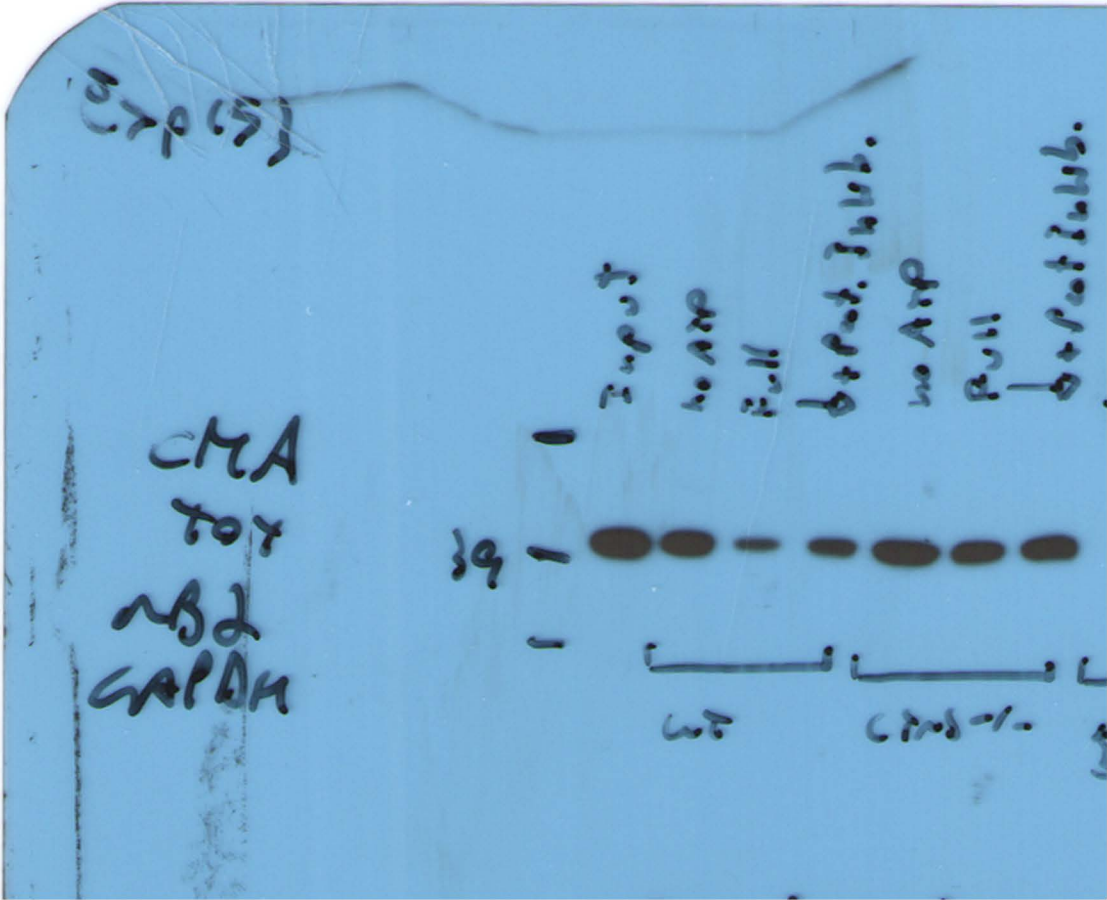

Supplement: Supplementary file 11 [file emmm0007-0158-sd11.pdf]

Figure 11C

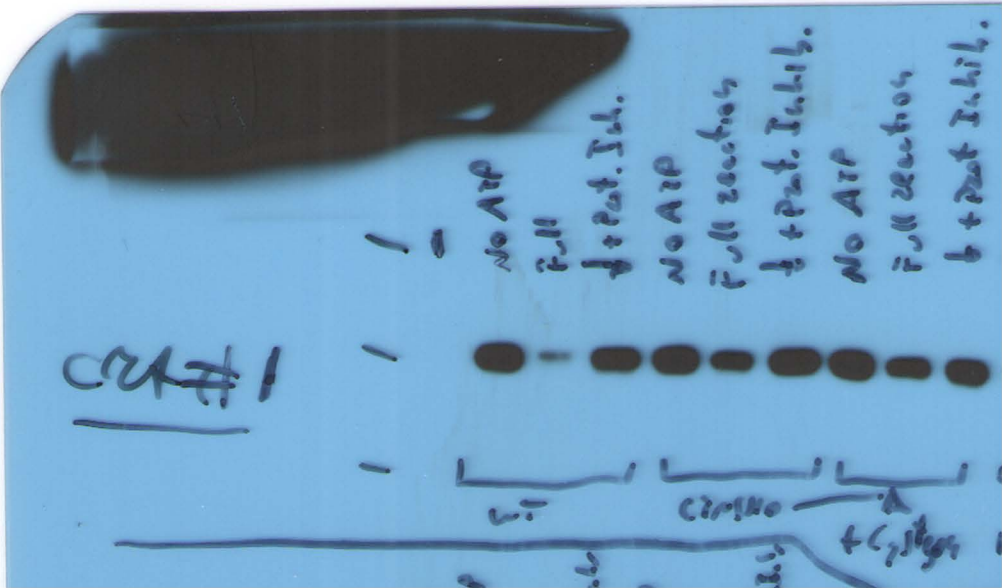

Supplement: Supplementary file 12 [file emmm0007-0158-sd12.pdf]
