# Supplementary material for: Impairment of chaperone-mediated autophagy leads to selective lysosomal degradation defects in the lysosomal storage disease cystinosis
Source: EMBO Mol Med. 2015 Jan 13;7(2):158–74. doi: 10.15252/emmm.201404223 (PMC4328646; doi:10.15252/emmm.201404223)
Supplement: Supplementary file 5 [file emmm0007-0158-sd5.pdf]

Supplementary information (SI)

Table of Contents

SI Materials and Methods

SI References

SI Figure legends

SI Materials and Methods

Constructs, transfections and transductions

The ptfLC3 vector encoding for GFP-RFP-LC3 was purchased from Addgene (Cat # 21074). The pCCL plasmid for lentiviral expression of GFP-CTNS was previously described (1). Generation of lentiviruses expressing GFP-CTNS and transduction in *Ctns-/-* cells were performed as described (1).

Gel electrophoresis and immunoblotting

Cells were lysed in lysis buffer containing 20 mM Tris (pH 7.4), 150 mM NaCl and 1% Triton X-100, in the presence of protease-inhibitor (Roche) and phosphatase-inhibitor (Calbiochem) cocktails. Following electrophoresis using 4-12% gradient gels (Life Sciences), proteins were transferred onto 0.45 μm nitrocellulose membranes and the membranes were incubated overnight in the indicated primary antibodies, then incubated with HRP-conjugated secondary antibodies. The blots were developed using SuperSignal West Pico, Dura or Femto chemiluminescence substrate systems (Thermo). Transferred proteins were visualized using Hyperfilm (Amersham Bioscience). The following antibodies were used for immunoblotting in this study: rat anti-LAMP1 (1D4B, Hybridoma Bank), rabbit anti-LAMP2a (Abcam), rabbit anti-actin (Sigma), rabbit anti-GAPDH (Gene Tex), mouse anti-GFP (Santa Cruz), and mouse anti-pS6K, rabbit anti-S6K, rabbit anti-active Caspase 3, rabbit anti-LC3B (Cell Signaling).

Immunofluorescence, immunohistochemistry and confocal analysis

Wild type and *Ctns-/-* cells were seeded on untreated number 1.5 borosilicate coverglass (Corning). Where indicated cells were starved as described above, then fixed with 4% paraformaldehyde, blocked with 1% BSA in PBS in the presence of 0.01% saponin. Samples were labeled with the indicated primary antibodies overnight at 4 °C in the presence of 0.01% saponin and 1% BSA.

Samples were washed and subsequently incubated with the appropriate combinations of Alexa Fluor (488, 568, 594, or 647)-conjugated donkey anti-rabbit, anti-rat, or anti-mouse secondary antibodies (Life Sciences).

For immunhistochemistry, tissues were fixed in Z-Fix (Anatech Ltd., MI) for 24 h and immediately processed with a tissue processor, embedded in paraffin, cut with a microtome and mounted on slides by standard procedures. Dewaxing and antigen retrieval were performed exactly as described previously (2). For immunofluorescence staining, samples were incubated in blocking buffer (1% bovine serum albumin [BSA], 0.01% saponin, PBS) for 1 h. The samples were incubated with primary antibodies overnight at 4°C, washed with PBS and labeled with Alexa Fluor-conjugated secondary antibodies. Nuclei were stained with 4′,6′-diamidino-2-phenylindole (DAPI), and samples were preserved in ProLong Gold antifade reagent (Molecular Probes) and kept at 4°C until analyzed. Samples were analyzed with a Zeiss LSM 710 laser scanning confocal microscope (LSCM) attached to a Zeiss Observer Z1 microscope at 21°C, using a 63× oil Plan Apo, 1.4-numerical-aperture objective. Images were collected using ZEN-LSM software and processed using ImageJ and Adobe Photoshop CS4. Analysis and quantification of GFP-LC3 puncta was achieved by ImagePro software. The following primary antibodies were used for IF in this study: rat anti-LAMP1 (1D4B, Santa Cruz) Goat anti-mLAMP1 (AF4320 R&D). anti-total LAMP2 (antibody ABL93 DSHB), rabbit anti-LAMP2a (Abcam Ab18528), rabbit anti-LAMP2a (Cuervo A, Dice J (2000) Journal of cell science 113 24**:** 4441-4450), rabbit anti-LC3B (Cell signaling), mouse anti-GFP (Santa Cruz), anti-Tim23 (BD biosciences), anti-Rab11a (Santa Cruz biotechnology) and anti-VAMP7 (SySy).

Lysosome fractionation and β-Hexosaminidase assay

The purification of the lysosomal fractions for CMA assays was performed by a modified protocol based on methods previously described (3, 4). Briefly, WT and *Ctns-/-* mice were starved for 24h, livers were collected, homogenized in Tris-buffered 0.25 M Sucrose (pH 7.4) in the absence of protease inhibitors and centrifuged at 6800 × *g* 15 min at 4 °C. After washing the pellet with 0.25 M sucrose, the combined supernatants were centrifuged at 17,000 × *g* 20 min at 4°C. The pellet, which contained the light mitochondria and lysosomes fraction, was then re-suspended in 0.25 M sucrose, and mitochondria were precipitated by incubation with 115 µM CaCl2 for 30 min. The

supernatants were then centrifuged 5000 × *g* for 10 min at 4°C to pellet the mitochondria. The supernatant, which contains the lysosomes, was collected and centrifuged again at 5000 × *g* for 10 min at 4 °C to ensure purity. The supernatant was retained and centrifuged at 17,000 × *g* for 20 min at 4 °C to pellet the lysosomes. The lysosomes were then re-suspended in reaction buffer (10 mM MOPS, pH 7.3, 0.25 M sucrose, 5.4 μM cysteine, 1 mM DTT). Lysosomal integrity was verified by comparative analysis of β-Hexosaminidase activity for intact lysosomes or 1% Triton X-100-lysed lysosomes. β-Hexosaminidase activity was measured using the membrane impermeant flourogenic substrate 4-methylumbelliferyl-N-acetyl-β-glucosaminide dehydrate (Sigma M2133) as previously described (5). Only lysosomes with an integrity of >85% were used in subsequent assays.

Analysis of cell death

Apoptosis was determined by evaluation of phosphatidylserine cell surface expression by flow cytometry using FITC-Annexin V staining. Cell death was measured by flow cytometry using Propidium Iodide (PI) staining (BD Pharmingen). After exposing cells with 50 μM H2O2 or 1 mM PQ for 4h, cells were harvested, washed in PBS and incubated for 15 min with either labeling compound. Stained cells were analyzed by flow cytometry in a FACSCalibur flow cytometer (BD Biosciences) and analyzed using FlowJo software.

Flow cytometry assays

Fibroblasts were labeled by incubation with LysoTracker for 3 min, following the manufacturer instructions (Life Sciences). The cells were washed, resuspended in PBS and immediately analyzed by flow cytometry. Fibroblasts were gated based on the specific labeling and data were collected using a FACSCalibur flow cytometer (BD Biosciences) and analyzed using FlowJo software.

Quantitative RT-PCR

RNA was isolated from wild type or *Ctns-/-* mouse fibroblasts using the RNeasy mini-kit for RNA purification (QIAGEN), which includes gDNA-eliminator columns. A total of 100 ng of RNA for each cell line was reverse-transcribed (RT) using iScript cDNA Synthesis Kit (Bio-Rad). Quantitative RT-PCR was performed using QuantiTect SYBR green PCR mix (Qiagen), with the

following primer mixes: m*Lamp2a*: GCAGTGCAGATGAAGACAAC – AGTATGATGGCGCTTGAGAC.

Degradation of long-lived proteins using radiolabeled amino acids

Degradation of long-lived proteins was measured essentially as described previously (6) with modifictions. WT or *Ctns*$^{-/-}$ cells were labeled with 11.7 µl of Easy tag Methionine L-^{35}S (cat # NEG709A, PerkinElmer) in growth medium containing 10% FCS. In these experiments, 11.7 µl of Easy tag Methionine L-^{35}S (10.25 mCi/ml) were added to cells to reach a of 0.010 mCi/ml. Cells were labeled for 48 hrs, washed with normal growth medium or starvation medium (normal growth medium without serum) and medium was replaced with the appropriate fresh media and cells were cultured for additional 15h. The acid-soluble radioactivity (AcsoR) in the medium was determined by precipitation with trichloroacetic acid (10%v/v). Total radioactivity incorporated into cellular proteins (CellR) was determined as the amount of acid-precipitable radioactivity in labeled cells immediately after washing. Proteolysis was measured as the rate AcsoRx100/(AcsoR+CellR).

Super resolution microscopy

STORM samples were prepared by labeling cells with anti-LAMP1 1D4B, Santa Cruz and Rabbit anti-LAMP2A (15 aa cytosolic tail, Cuervo and Dice, 1996), primary antibodies and Alexa-647 anti-Rat and Alexa-488 anti-Rabbit secondary antibodies. Samples were suspended in freshly prepared STORM buffer [50 mM Tris pH 8.0, 10 mM NaCl, 10 % Glucose, 0.1 M Mercaptoethanolamine (Cysteamine Sigma-Aldrich), 56 U/ml Glucose Oxidase (from Aspergillus niger, Sigma-Aldrich), and 340 U/ml Catalase (from bovine liver, Sigma-Aldrich)], and imaged on a Nikon Ti super resolution microscope. Samples were imaged using a 100X 1.49 NA Apo TIRF objective either with or without TIRF illumination. Images were collected on an ANDOR IXON3 Ultra DU897 EMCCD camera using the multi-color continuous mode setting in the Nikon Elements software. Power on the 488 nm and 647 nm lasers was adjusted to enable collection of between 50 and 300 molecules per 256x256 camera pixel frame at appropriate threshold settings for each channel. Collection was stopped after a sufficient number of frames were collected (usually yielding 1-2 million molecules), and the super resolution images were reconstructed with the Nikon STORM software.

SI References

1. Harrison F, *et al.* (2013) Hematopoietic stem cell gene therapy for the multisystemic lysosomal storage disorder cystinosis. *Molecular therapy: the journal of the American Society of Gene Therapy* 21(2):433-444.

2. Robertson D, Savage K, Reis-Filho J, & Isacke C (2008) Multiple immunofluorescence labelling of formalin-fixed paraffin-embedded (FFPE) tissue. *BMC cell biology* 9:13.

3. Malkus K & Ischiropoulos H (2012) Regional deficiencies in chaperone-mediated autophagy underlie α-synuclein aggregation and neurodegeneration. *Neurobiology of disease* 46(3):732-744.

4. Kaushik S & Cuervo A (2008) Chaperone-mediated autophagy. *Methods in molecular biology (Clifton, N.J.)* 445:227-244.

5. Rodríguez A, Webster P, Ortego J, & Andrews N (1997) Lysosomes behave as Ca2+-regulated exocytic vesicles in fibroblasts and epithelial cells. *The Journal of cell biology* 137(1):93-104.

6. Massey A, Kaushik S, Sovak G, Kiffin R, Cuervo A (2006a) Consequences of the selective blockage of chaperone-mediated autophagy. Proceedings of the National Academy of Sciences of the United States of America 103: 5805-5810

7. Choy et al. (2012) The *Legionella* Effector RavZ Inhibits Host Autophagy Through Irreversible Atg8 Deconjugation Science: Vol. 338 no. 6110 pp. 1072-1076

8. Lupfer et al 2013 Receptor interacting protein kinase 2–mediated mitophagy regulates inflammasome activation during virus infection *Nature Immunology* 2013: 14, 480–488

SI Figure legends

Fig. S1. *Macroautophagic flux is increased in Ctns-/- fibroblasts and bone marrow-derived macrophages (BMDM)*

A. LC3B-II turnover was measured in WT and *Ctns-/-* fibroblasts by Western blot (WB) under resting conditions (-), withdrawal of both amino acids and serum (Aa/Ser. Starv.), or withdrawal of serum only (Ser. Starv.), in the presence or absence of 100 nM Bafilomycin A (BafA) for the indicated time points. Data are representative of 3 different experiments with similar results. B.

LC3B-II turnover was analyzed by WB using bone marrow macrophages (BMDM) derived from WT or *Ctns*$^{-/-}$ mice. Experiments were performed under similar conditions as in A, except that time points were adapted to visualize the slower response characteristic of macrophages. The only band visible in B corresponds to LC3-II as determined by MW. The presence of a single prominent LC3-II band in bone marrow macrophages have been shown in several reports (7, 8).

Fig. S2. *Ctns*$^{-/-}$ *cells have increased number of lysosomes*

A, Cells were labeled with Lysotracker (pink and blue lines) or left unlabeled (grey and green lines) and analyzed by flow cytometry as described under "SI Materials and Methods". Data is representative of three independent experiments. B, Endogenous LAMP1 localization analyzed by confocal microscopy showed increased LAMP1 staining in circular structures in *Ctns*$^{-/-}$ cells compared to wild type (WT) mouse fibroblasts, indicating increased amount of lysosomes in these cells. Scale bars=10 μm.

Fig. S3. *WT and Ctns*$^{-/-}$ *cells have similar Lamp2a RNA expression levels*

Wild type (WT) and *Ctns*$^{-/-}$ fibroblasts were left untreated or serum starved (Ser. Starv.) for 20h to activate CMA, then harvested for quantitative RT-PCR analysis. *Lamp2a* expression (corrected for expression of the control gene *L32*) was similar in WT and *Ctns*$^{-/-}$ cells in all conditions tested. Results are mean ± SEMs.

Fig. S4. *Lysosomal quality control*

A, Western blot analyses of subcellular structural markers in several fractions obtained from the purification of WT or *Ctns*$^{-/-}$ lysosomes. PNS, post-nuclear supernatant; Cyt, cytosol; Mit, Mitochondrial fraction and Lys, lysosomal fraction. B, Lysosomal integrity was determined by evaluating β-hexosaminidase activity in either untreated (UN) or detergent-treated lysosomal fractions (D).
