# Supplementary material for: Impairment of chaperone-mediated autophagy leads to selective lysosomal degradation defects in the lysosomal storage disease cystinosis
Source: EMBO Mol Med. 2015 Jan 13;7(2):158–74. doi: 10.15252/emmm.201404223 (PMC4328646; doi:10.15252/emmm.201404223)
Supplement: Supplementary file 7 [file emmm0007-0158-sd7.pdf]

Impairment of chaperone-mediated autophagy leads to selective lysosomal degradation defects in the lysosomal storage disease cystinosis

Gennaro Napolitano, Jennifer L. Johnson, Jing He, Celine J Rocca, Jlenia Monfregola, Kersi Pestonjamasp, Stephanie Cherqui and Sergio D. Catz

Corresponding author: Sergio Catz, The Scripps Research Institute

Review timeline:

Submission date:	06 May 2014
Editorial Decision:	21 June 2014
Revision received:	23 October 2014
Editorial Decision:	24 November 2014
Revision received:	07 December 2014
Accepted:	09 December 2014

Transaction Report:

(Note: With the exception of the correction of typographical or spelling errors that could be a source of ambiguity, letters and reports are not edited. The original formatting of letters and referee reports may not be reflected in this compilation.)

Editor: Roberto Buccione

1st Editorial Decision 21 June 2014

Thank you for the submission of your manuscript to EMBO Molecular Medicine. We are sorry that it has taken longer than usual to get back to you on your manuscript. In this case we experienced unusual difficulties in securing three appropriate reviewers and then obtaining their evaluations in a timely manner.

You will see that the three Reviewers express several, partially overlapping concerns that prevent us from considering publication at this time. My overall impression is that all Reviewers express dissatisfaction (to varying degrees) with the experimental support provided. This includes missing controls, need for further experimentation to support the conclusions and over-interpretation.

I would like to just mention a few specific points without dwelling into much detail, as the evaluations are quite detailed and self-explanatory.

Reviewer 1 notes the further evidence is required to demonstrate the autophagy effects in cystinosin-deficient cells, including monitoring of longer-lived proteins and would also like to see whether LAMP2 or LAMP2A glycosylation is altered. S/he is also is concerned that images often do not support the conclusions. Reviewer 1 lists other very important issues.
Reviewer 2, while recognising the interest of your work, is also quite concerned that the data do not necessarily match the conclusions.

Reviewer 3 notes, as does Reviewer 1, that the data provided do not reliably assign the key role in CMA to LAMP2A. S/he is also quite concerned that the CMA assays are of insufficient quality and not convincing. This Reviewer also lists other very important issues.

I would like to add that as far as we could determine, the figures provided do not have technical (i.e. not experimentally related) issues.

Considered all the above, while publication of the paper cannot be considered at this stage, we would be prepared to consider a substantially revised submission, with the understanding that the Reviewers' concerns must be fully addressed with additional experimental data where appropriate and that acceptance of the manuscript will entail a second round of review.

Please note that it is EMBO Molecular Medicine policy to allow a single round of revision only and that, therefore, acceptance or rejection of the manuscript will depend on the completeness of your responses included in the next, final version of the manuscript.

As you know, EMBO Molecular Medicine has a "scooping protection" policy, whereby similar findings that are published by others during review or revision are not a criterion for rejection. However, I do ask you to get in touch with us after three months if you have not completed your revision, to update us on the status. Please also contact us as soon as possible if similar work is published elsewhere.

I look forward to seeing a revised form of your manuscript as soon as possible.

***** Reviewer's comments *****

Referee #1 (Remarks):

The authors start by showing that LC3B protein levels are increased in cystinosin-deficient cells. Since the LC3-positive vesicles seem to become normally acidified, and LC3 turnover appears normal, the authors conclude that the formation of autophagosomes is increased in the cystinosin-deficient cells. mTOR activity is not affected in these defective cells. However, the authors show that the levels and localization of LAMP2A are altered, and that chaperone-mediated autophagy is not functional due to defective translocation of substrates into the lysosome lumen. Increased macroautophagy is explained to be due to the block in chaperone-mediated autophagy. LAMP2A level and localization are rescued by ectopic expression of cystinosin and by lysosomal inhibitors, but not by cysteamine, which is able to reduce the lysosomal accumulation of cystine. Finally the authors also show that the cystinosin-deficient cells are more susceptible to oxidative stress than wild type cells.

The manuscript is well written and the findings are very interesting. However, some important controls are missing, and more experimental data is needed to support the conclusions.

1. Nomenclature of LAMP2 isoforms. The authors should follow the nomenclature and the typing recommendation given in Traffic 2005, 6:1058-1061 (LAMP2A instead of LAMP2a). Because of the former confusion on mouse vs. human nomenclature of LAMP2 isoforms (see the Traffic article), the authors should state the sequence of the mouse LAMP2A C-terminus. This point is important concerning the RT-PCR experiment on page 8.

2. The authors conclude on page 7 that increased numbers of autophagosomes in the deficient cells are due to increased autophagic flux. However, the data in Fig. 2D could be interpreted differently. Comparing LC3-II levels on lanes 2 and 4 (WT) and 6 and 8 (deficient cells) indicate that during the recovery, LC3 degradation is slower in the cystinosin-deficient cells compared to WT cells.

3. The authors should do additional experiments to convince the readers that autophagic flux in increased in the cystinosin-deficient cells. SQSTM1 levels should be monitored using the conditions shown in Fig. 2 and Fig. S1. The authors should also measure the degradation of long-lived proteins using metabolic labeling with a radioactive amino acid.

4. Fibroblasts express also LAMP2B. Protein levels and localization of total LAMP2 should also be shown (Fig. 3). An excellent antibody against total LAMP2 (ABL93) is available from the same source (DSHB) as the anti-LAMP1 used in this manuscript. Does total LAMP-2 colocalize with LAMP1 in the cystinosin-deficient cells?

5. Did the authors check the specificity of the commercial anti-LAMP2A they are using? What is the molecular weight of the LAMP2A band?

6. Glycosylation is thought to be important in protecting lysosomal proteins against degradation. Is the glycosylation of LAMP2 or LAMP2A altered in cystinosin-deficient cells?

7. On page 10, the authors conclude that the cystinosin-deficient lysosomes still bind the CMA substrate, despite the data showing that these cells have only low level of LAMP2A protein. This is surprising, since LAMP2A is thought to be the receptor that binds the CMA substrates on the lysosomal surface.

8. The authors describe the LAMP2A localization in cystinosin-deficient cells as 'reticular distribution'. Is LAMP2A colocalizing with markers of endoplasmic reticulum, or other organelle markers? Where is LAMP2A (and total LAMP2) localized in the cystinosin-deficient cells?

9. On page 11, the authors write: "CTNS itself may be necessary for proper LAMP2A localization." This is in conflict with Fig. S4, which shows that lysosomal inhibitors alone are able to rescue the correct localization of LAMP2A in cystinosin-deficient cells.

10. Does LAMP2 or LAMP2A colocalize and/or interact with CTNS in wild type cells?

11. Fig. 1B, Fig. 1C. There are no LC3B or other vesicles visible in these images. Therefore, it is hard to tell how reliable the quantifications are.

12. No red-only vesicles are visible in Fig. 2A. If there were no red-only vesicles in the samples, then how reliable are the quantifications shown in Fig. 2B?

13. Loading control (such as LAMP1 level by Western blot) should be included in Fig. 4B and 4D.

14. Fig. 2E. The authors should use ectopic expression of GFP only as an additional control.

15. Figure S4 is very interesting and important. It is astonishing that it has been hidden in the Supplement.

16. It would be very helpful for the readers if the authors would explain their experimental model system before starting to explain the results (page 5, first paragraph).

17. Fig. S1B. There is only one band for LC3. Is this LC3-I or LC3-II?

Referee #2 (Comments on Novelty/Model System):

This is an interesting manuscript showing that the lysosomal storage disease cystinosis, which involves inability to transport cystine out of lysosomes, is accompanied by a defect in chaperone-mediated autophagy. This defect can be rescued by lentiviral transduction with the cystinosin gene but not by clearance of the accumulated cystine. This finding is quite novel, and has important implications for understanding the pathophysiology of the disease and for developing therapy that would address the autophagy defect.

There is a problem with the immunofluorescence images. They were clearly defective in the pdf that contained text and figures, and these comments are based on the figures in the source files. The main point of the paper is summarized in Fig 6, which is easy to understand and of high quality. Fig 3, which has the same information as the first two rows of Fig 6, is of lower quality because the magnified LAMP1 panel is out of focus, but is acceptable. But in Fig. 1, I can see green dots almost exclusively in the bottom right panel; the bar graphs do not correspond to what I see in the images. And in Fig. 2, I can barely see the colors. I assume that the problem with Fig 1 and 2 is in the transmission of the very large files from the authors to the journal to my computer. Perhaps the journal can assist the authors in solving this problem.

Referee #2 (Remarks):

This is an interesting and important manuscript which shows that cystinosis is not merely a disease of lysosomal storage of cystine, but also involves a defect of chaperone-mediated autophagy. The authors go through a number of possibilities, and show that the defect in CMA is independent of the lysosomal storage.

The paper is generally well written, and a few minor suggestions are listed below. But the confocal images (Fig 1 and 2) need attention because at the present time they do not correspond to the bar graphs derived from them.

The following are minor suggestions:
a. Many sentences begin with "Importantly". Usually, that term contributes nothing to the meaning and can be annoying to the reader.
b. Use of cysteamine is not necessarily "substrate reduction". It's finding an alternate pathway to transport of cystine out of the lysosome.
c. P. 4 and elsewhere - "complementary treatments to substrate depletion " should say "treatments complementary to substrate depletion..."
d. Fig. 6 is placed out of order on p. 8, end of first paragraph. It would be better to omit that sentence.
e. The last paragraph of the discussion is redundant with the paragraph preceding it and can be omitted.

Referee #3 (Remarks):

The manuscript by Napolitano and colleagues deals with aspects of cystinosis, which is a lysosomal storage disease. The authors addressed the impact of the CTNS deficiency on macroautophagy, chaperone-mediated autophagy and lysosomal overload. Aspects of this work are highly relevant, however, the reviewer has several concerns about experimental approaches and the interpretation of the data, which need to be clarified in a thorough revision.

Fig.1: The LC3 signal is hardly detectable in the IF images, while the DNA staining is very prominent. The images should either be replaced, or the authors need provide blow-ups.

A major part of this work addresses the so-called chaperone-mediated autophagy. In this context, the authors state their own previous work (Johnson et al., 2013) claiming that the size and number of lysosomes is increased in cystinotic cells. However, the earlier work contains no data on the altered size of lysosomes, which is further not recognizable from the IF in figures 3 or 6 of the manuscript. The authors document increased Lamp-1 expression in cystinotic cells by westernblots, and show an increased Lamp-1 staining in figure S2. However, in figure 1 this increased staining of cystinotic cells is not at all visible. Further, the Lamp-1 expression for kidney is not documented. Also the authors show western of "lysosomes". What was loaded? Equal protein amounts of purified lysosomes? Here, Lamp-1 levels are equal to the controls. In addition, the quality of the lysosomal preparations is not documented at all.

The authors show mis-localisation of Lamp-2a in various figures. However, upon inhibition of lysosomal degradation they state "...that defective expression and localization of LAMP2a in cystinotic cells is due to its excessive lysosomal degradation:" So obviously there is no mis-localisation, because otherwise the protein would not be accessible to lysosomal degradation. It may well be that Lamp-1 and Lamp-2a are partially segregated in the lysosomal membrane. The authors state several possibilities for the surprisingly selective effect of the protease inhibitor treatment, however to the reviewers knowledge there is no convincing mechanistic insight presented so far.
In this context the reviewer likes to mention the critical importance of unequivocal detection of Lamp-2a as the predicted key player in CMA. The authors used a commercially available antibody against Lamp-2a, which was raised against a region of more than 50 residues. There is sequence variation within this region of the three Lamp-2a isoforms, however it remains unclear to me where the isoform specificity of the used antibody is documented.

Fig. 4: Assays for CMA: The figure shows the workflow for a lysosome preparation, however the quality of the preparation is not documented (see above). In B, a western is presented to document the CMA mediated degradation of GAPDH. In the full reaction (lane3), no remaining GAPDH is visible. However, the statistics presented in C document an average protease resistant fraction of around 40%. Thus, B shows a rather extreme experiment, but not a representative example?? I am puzzled by the figure legend, because the differences between assays in B and E are not indicated. Were lysosomes in B not separated by centrifugation? In E, the authors claim that the data shows GAPDH bound to the lysosomal membrane and the "internalized" fraction. The designation of this fraction is inappropriate, because the process is thought to occur via translocation through the membrane. Further, the controls are missing. The input amount of GAPDH is missing, as well as GAPDH incubated without lysosomes. For appropriate analysis, the authors would need to show the levels of GAPDH in the supernatant after centrifugation. The respective M&M section is unclear: What is the difference between a CMA assay and an "uptake assay"? Were lysosomes only pelleted for the latter assay? As displayed, the data is insufficient to draw any conclusion and the interpretation of the data on page 10 is a massive over-interpretation.
Discussion: The authors state defective degradation of CMA substrates, but they only analysed a single one.

The authors speak about "...the decreased expression of LAMP2a at the lysosomal membrane", which is misleading. They probably mean the lysosomal content of Lamp-2a, or the detectable levels of the protein. Further in the text they present a Ñhypothesis" about a direct role of CTNS in the regulation of the ÑCMA receptor complex". This is not a hypothesis, but pure speculation. Finally, at the very end of the discussion the statements of the authors are redundant.
In summary, I see massive deficits in the analysis of CMA and its interpretation. Furthermore, it remains unclear, whether mis-localisation of Lamp-2a occurs at all. Both issue require adequate attention in a revision of the manuscript.

1st Revision - authors' response 23 October 2014

Referee #1 (Remarks):

The authors start by showing that LC3B protein levels are increased in cystinosin-deficient cells. Since the LC3-positive vesicles seem to become normally acidified, and LC3 turnover appears normal, the authors conclude that the formation of autophagosomes is increased in the cystinosin-deficient cells. mTOR activity is not affected in these defective cells. However, the authors show that the levels and localization of LAMP2A are altered, and that chaperone-mediated autophagy is not functional due to defective translocation of substrates into the lysosome lumen. Increased macroautophagy is explained to be due to the block in chaperone-mediated autophagy. LAMP2A level and localization are rescued by ectopic expression of cystinosin and by lysosomal inhibitors, but not by cysteamine, which is able to reduce the lysosomal accumulation of cystine. Finally the authors also show that the cystinosin-deficient cells are more susceptible to oxidative stress than wild type cells.
The manuscript is well written and the findings are very interesting. However, some important controls are missing, and more experimental data is needed to support the conclusions.

1. Nomenclature of LAMP2 isoforms. The authors should follow the nomenclature and the typing recommendation given in Traffic 2005, 6:1058-1061 (LAMP2A instead of LAMP2A). Because of the former confusion on mouse vs. human nomenclature of LAMP2 isoforms (see the Traffic article), the authors should state the sequence of the mouse LAMP2A C-terminus. This point is important concerning the RT-PCR experiment on page 8.

We thank the reviewer for the suggestion. The nomenclature for LAMP2A has been corrected throughout the manuscript and figures.

In addition, we have now stated the sequence of the mouse LAMP2A C-terminus on page 9, end of first paragraph. *LAMP2A* (Ref Seq NM_001017959).

2. The authors conclude on page 7 that increased numbers of autophagosomes in the deficient cells are due to increased autophagic flux. However, the data in Fig. 2D could be interpreted differently. Comparing LC3-II levels on lanes 2 and 4 (WT) and 6 and 8 (deficient cells) indicate that during the recovery, LC3 degradation is slower in the cystinosin-deficient cells compared to WT cells.

We have incorporated the reviewer's analysis to the interpretation of our results. In Page 7, 1st paragraph, line 10 we wrote: "Although the greater accumulation of LC3B-II observed after nutrient recovery in *Ctns*$^{-/-}$ cells compared to wild type cells (Fig. 2D, lanes 4 and 8) may suggest that degradation is slower in *Ctns*$^{-/-}$ cells under recovery experimental conditions, the observation that starvation itself induces a prompt reduction in the levels of LC3BII in *Ctns*$^{-/-}$ cells (Fig. 2D

lanes 5 and 6, Fig. S1A and B lanes 6 and 7) argues against a marked defective degradation phenotype in these cells (also shown in Fig. 3).

We also wrote (p7, 1st paragrapg, line 21): "Altogether, these data show that autophagosome maturation is not impaired in cystinotic cells, and that *Ctns*$^{-/-}$ cells are characterized by increased numbers of autophagosomes but a fully functional autophagic flux."

3. The authors should do additional experiments to convince the readers that autophagic flux in increased in the cystinosin-deficient cells. SQSTM1 levels should be monitored using the conditions shown in Fig. 2 and Fig. S1. The authors should also measure the degradation of long-lived proteins using metabolic labeling with a radioactive amino acid.

We have now performed these experiments and have incorporated the results in the new Figure 3. SQSTM1 levels decreased under starvation conditions in both WT and *Ctns*-/- cells (new Fig. 3A-C), again suggesting that the autophagic degradation machinery in *Ctns*-/- cells is intact. These was further confirmed by analysis of long-lived protein degradation using radiolabeled amino acids (new Figure 3D), which shows similar levels of proteolysis for both WT and *Ctns*-/- cells. In addition, the levels of proteolysis were similar to those described in the literature in similar assays performed in cells with compromised CMA (for example, see Massey et al. PNAS 2006). Overall, our data from Fig. 2 and new Fig. 3 rule out major defects in autophagosome maturation or degradation. Our data also show increased number of autophagosomes in *Ctns*-/- cells which is most likely a compensatory mechanism induced by defective CMA similar to that observed in LAMP2-deficient cells (Massey et al, PNAS 2006). Altogether, we describe the dynamic characteristics of autophagosomes in *Ctns*-/- cells that include high basal number, accumulation upon inhibition of fusion (functional maturation) and responsiveness to starvation (functional degradation).

4. Fibroblasts express also LAMP2B. Protein levels and localization of total LAMP2 should also be shown (Fig. 3). An excellent antibody against total LAMP2 (ABL93) is available from the same source (DSHB) as the anti-LAMP1 used in this manuscript. Does total LAMP-2 colocalize with LAMP1 in the cystinosin-deficient cells?

Using an antibody directed to the carboxy-terminal domain of LAMP2B, we analyzed the localization of LAMP2B in *Ctns*-/- and WT cells. Our data (new Figure 5C) show that LAMP2B colocalizes with LAMP1 in both *Ctns*-/- and WT cells. In addition, some colocalization between total LAMP2 and LAMP1 was observed in *Ctns*-/- cells when the suggested ABL93 antibody was used (Fig. 5D). This is expected because of the lower expression of LAMP2A and the correct localization of LAMP2B in Ctns-/-. A subpopulation of total LAMP2-positive but LAMP1- negative structures was also observed in *Ctns*-/- cells with

ABL93 antibody, most likely consisting of LAMP2A, further supporting that LAMP2A is mislocalized in these cells.

5. Did the authors check the specificity of the commercial anti-LAMP2A they are using? What is the molecular weight of the LAMP2A band?

We have now performed experiments with a different LAMP2A antibody raised against the twelve amino acids of the cytosolic region of rat LAMP2A largely validated to recognize mouse LAMP2A but not other LAMP2 isoforms (Cuervo and Dice, Journal of cell science 113 Pt 24**:** 4441-4450). Using this antibody, we confirmed the MW of LAMP2A, as well as its lower expression in *Ctns-/-* cells (new Figure 4D). The MW is about 95 KDa which is the expected MW for LAMP2A.

6. Glycosylation is thought to be important in protecting lysosomal proteins against degradation. Is the glycosylation of LAMP2 or LAMP2A altered in cystinosin-deficient cells?
This is an important question that requires further examination. In this manuscript we present data that suggest both partial mislocalization of LAMP2A and increased degradation of the protein that reaches the lysosome. These defects that *a priori* may be interpreted as independent findings, may have a common cause in, for example, deficient LAMP2A glycosylation. Our new data showing low colocalization of LAMP2A with VAMP7 and increased colocalization with Rab11a (new Fig. 6), support that LAMP2A trafficking may be altered, a process that is believed to be dependent on LAMP glycosylation and on other post-translational modifications. However, we believe that the study of post-translational modifications of LAMP2A requires extensive analyses that are beyond the scope of this manuscript that already contains extensive novel data.

7. On page 10, the authors conclude that the cystinosin-deficient lysosomes still bind the CMA substrate, despite the data showing that these cells have only low level of LAMP2A protein. This is surprising, since LAMP2A is thought to be the receptor that binds the CMA substrates on the lysosomal surface.
Previous studies suggested that binding of CMA substrates to LAMP2A does not exclusively depend on the amount of receptor but also on other parameters such as sublocalization of LAMP2A at microdomains or its state as a monomer/ multimer. Based on those studies, it is likely that a residual amount of LAMP2A on lysosomal membranes is prominently in a monomeric state, which allows binding but not translocation of CMA substrates. Thus if the number of monomeric LAMP2A on lysosomal membranes is similar in WT compared to KO cells, this would explain similar binding and defective translocation. Our new high resolution microscopy data suggest that the localization of LAMP2A at distinct lysosomal subdomains in Ctns-/- is plausible (new Fig. 5B).
Having said that, given that binding experiments raised questions by 2 different reviewers, the binding/translocation assay (old Figure 4E) and associated text has been removed from the manuscript.

8. The authors describe the LAMP2A localization in cystinosin-deficient cells as 'reticular distribution'. Is LAMP2A colocalizing with markers of endoplasmic reticulum, or other organelle markers? Where is LAMP2A (and total LAMP2) localized in the cystinosin-deficient cells?

We have performed new immunofluorescence analyses of LAMP2A in Ctns-/- cells (new Figure 6). We found that LAMP2A does not colocalizes with ER markers in Ctns-/- cells ruling out entrapment in the ER (new Fig. 6A). However, we found that LAMP2A colocalizes with VAMP7 in wild type cells but much less in Ctns-/- cells (Fig. 6B). Since VAMP7 mediates direct Golgi to lysosome translocation of lysosomal membrane associated proteins (Pols et al, 2013), our new data suggest possible trafficking defects of LAMP2A in addition to the degradative abnormalities of LAMP2A in these cells. This is further supported by the increased colocalization of LAMP2A with the trafficking GTPase Rab11 in Ctns-/- cells but not WT cells. (Fig. 6C).

9. On page 11, the authors write: "CTNS itself may be necessary for proper LAMP2A localization". This is in conflict with Fig. S4, which shows that lysosomal inhibitors alone are able to rescue the correct localization of LAMP2A in cystinosin-deficient cells.

The sentence was removed

10. Does LAMP2 or LAMP2A colocalize and/or interact with CTNS in wild type cells?

This is an important question that is being pursued in our lab. At this time, we cannot confirm or rule out a direct effect of CTNS on LAMP2A stabilization. In Page 15, end of 2nd paragraph we wrote “Our finding that CTNS expression, but not reduction of lysosomal overload, increases the detectable levels of LAMP2A at the lysosomal membrane (Fig. 10) supports the idea that CTNS may help stabilize LAMP2A at the lysosomal membrane either directly or indirectly”. Interaction of CTNS with LAMP2A and colocalization by super resolution microscopy are currently being investigated in our lab.

11. Fig. 1B, Fig. 1C. There are no LC3B or other vesicles visible in these images. Therefore, it is hard to tell how reliable the quantifications are.

We are very sorry for this inconvenient. Despite having uploaded high-quality images on the EMM website, the quality was affected during the automatic conversion of original files to a single pdf, therefore images were not displayed properly in the single pdf. We have discussed this issue with the Editorial staff and the images should now be of adequate resolution. In addition, there should be individual figure files (separate files from the pdf) at high resolution to facilitate the reviewer’s work.

12. No red-only vesicles are visible in Fig. 2A. If there were no red-only vesicles in the samples, then how reliable are the quantifications shown in Fig. 2B?

The reason for this inconvenient has been discussed in the answer to question 11. Red-only vesicles should now be visible.

13. Loading control (such as LAMP1 level by Western blot) should be included in Fig. 4B and 4D.

The loading control (LAMP1) is now shown in new figure 8B.

14. Fig. 2E. The authors should use ectopic expression of GFP only as an additional control.

Data not shown

15. Figure S4 is very interesting and important. It is astonishing that it has been hidden in the Supplement.

We agree with the reviewer and figure S4 is now incorporated into the main body (New Figure 7).

16. It would be very helpful for the readers if the authors would explain their experimental model system before starting to explain the results (page 5, first paragraph).

Thanks. This is now addressed in page 5, first paragraph. We now wrote "In this work, to better understand the regulation of autophagy in cystinosis, we analyze macroautophagic flux and chaperon mediated autophagy using *Ctns*$^{-/-}$ mice, *Ctns*$^{-/-}$-GFP-LC3 transgenic mice and *Ctns*$^{-/-}$ -derived embryonic fibroblasts."

17. Fig. S1B. There is only one band for LC3. Is this LC3-I or LC3-II?
The only band visible by MW represents LC3-II. LC3-I/II ratios were described to vary among cell lines and bone marrow macrophages have been shown to have a prominent LC3-II band in several reports; for example, Choy et al. Science 2012: Vol. 338 no. 6110 pp. 1072-1076 and Lupfer et al. (2013) *Nature Immunology* 14, 480–488.
This is now clarified in the Figure legend S1.

Referee #2 (Comments on Novelty/Model System):

This is an interesting manuscript showing that the lysosomal storage disease cystinosis, which involves inability to transport cystine out of lysosomes, is

accompanied by a defect in chaperone-mediated autophagy. This defect can be rescued by lentiviral transduction with the cystinosin gene but not by clearance of the accumulated cystine. This finding is quite novel, and has important implications for understanding the pathophysiology of the disease and for developing therapy that would address the autophagy defect.

There is a problem with the immunofluorescence images. They were clearly defective in the pdf that contained text and figures, and these comments are based on the figures in the source files. The main point of the paper is summarized in Fig 6, which is easy to understand and of high quality. Fig 3, which has the same information as the first two rows of Fig 6, is of lower quality because the magnified LAMP1 panel is out of focus, but is acceptable. But in Fig. 1, I can see green dots almost exclusively in the bottom right panel; the bar graphs do not correspond to what I see in the images. And in Fig. 2, I can barely see the colors. I assume that the problem with Fig 1 and 2 is in the transmission of the very large files from the authors to the journal to my computer. Perhaps the journal can assist the authors in solving this problem.

We are very sorry for this inconvenient. Despite having uploaded high-quality images on the EMM website, the quality was affected during the automatic conversion of original files to a single pdf, therefore images were not displayed properly in the single pdf. We have discussed this issue with the Editorial staff and the images should now be of adequate resolution. In addition, there should be individual figure files (separate files from the pdf) at high resolution available to facilitate the reviewer's work.

Referee #2 (Remarks):

This is an interesting and important manuscript which shows that cystinosis is not merely a disease of lysosomal storage of cystine, but also involves a defect of chaperone-mediated autophagy. The authors go through a number of possibilities, and show that the defect in CMA is independent of the lysosomal storage.

The paper is generally well written, and a few minor suggestions are listed below. But the confocal images (Fig 1 and 2) need attention because at the present time they do not correspond to the bar graphs derived from them.
The high quality images should now be available.

The following are minor suggestions:
a. Many sentences begin with "Importantly". Usually, that term contributes nothing to the meaning and can be annoying to the reader.
We have removed the word “importantly” from several sentences throughout the manuscript.

b. Use of cysteamine is not necessarily "substrate reduction". It's finding an alternate pathway to transport of cystine out of the lysosome.
We have replaced "substrate reduction" for “substrate-reduction and lysosomal overload-decreasing” therapies when describing general aspects of LSDs and for “lysosomal overload-decreasing” when discussing cystinosis in particular.
c. P. 4 and elsewhere - "complementary treatments to substrate depletion " should say "treatments complementary to substrate depletion..."
We thank the reviewer for this suggestion. The sentence has been changed.

d. Fig. 6 is placed out of order on p. 8, end of first paragraph. It would be better to omit that sentence.
The sentence has been deleted.

e. The last paragraph of the discussion is redundant with the paragraph preceding it and can be omitted.
This has been corrected.

Referee #3 (Remarks):

The manuscript by Napolitano and colleagues deals with aspects of cystinosis, which is a lysosomal storage disease. The authors addressed the impact of the CTNS deficiency on macroautophagy, chaperone-mediated autophagy and lysosomal overload. Aspects of this work are highly relevant, however, the reviewer has several concerns about experimental approaches and the interpretation of the data, which need to be clarified in a thorough revision.

Fig.1: The LC3 signal is hardly detectable in the IF images, while the DNA staining is very prominent. The images should either be replaced, or the authors need provide blow-ups.
We are very sorry for this inconvenient. Despite having uploaded high-quality images on the EMM website, the quality was affected during the automatic conversion of original files to a single pdf, therefore images were not displayed properly in the single pdf. We have discussed this issue with the Editorial staff and the images should now be of adequate resolution. In addition, there should be individual figure files (separate files from the pdf) at high resolution available to facilitate the reviewer’s work.

A major part of this work addresses the so-called chaperone-mediated autophagy. In this context, the authors state their own previous work (Johnson et al., 2013) claiming that the size and number of lysosomes is increased in cystinotic cells. However, the earlier work contains no data on the altered size of lysosomes, which is further not recognizable from the IF in figures 3 or 6 of the manuscript.

The increased size of lysosomes in cystinotic cells was shown in Figure 3G (columns 2 and 5 “Lyso”) in our previous publication: Johnson et al., Mol Cell Biol 2013 vol. 33 no. 15 2950-2962. In that work, WT and cystinotic cells were analyzed by electron microscopy and lysosomal volume was quantified by stereology. The differences might not be appreciated by confocal microscopy analysis due to its limited resolution. This is now clarified on page 9, 1st paragraph, line 6.

The authors document increased Lamp-1 expression in cystinotic cells by western blots, and show an increased Lamp-1 staining in figure S2. However, in figure 1 this increased staining of cystinotic cells is not at all visible.
Figure 1 does not show LAMP1, but LC3. The image should be now of high quality in both pdf and individual files.

Further, the Lamp-1 expression for kidney is not documented.
This is now included in new Fig. 4B. Again, showing that LAMP1 is increased in Ctns-/- cells and tissues.

Also the authors show western of "lysosomes". What was loaded? Equal protein amounts of purified lysosomes? Here, Lamp-1 levels are equal to the controls.

Equal protein of purified lysosomes were loaded for WB analyses presented in old Fig. 3C (now Fig. 4C) and in new Figure 4D. The increased levels of LAMP1 in Ctns-/- cells reflect the increased lysosomal number in these cells. WT and Ctns-/- purified lysosomes show similar levels of LAMP1 expression/protein unit.

In addition, the quality of the lysosomal preparations is not documented at all.
This is now documented (included in Supplementary Figure S4). Please notice that not only the lysosome preparation is not “contaminated” with other organelles (Fig. S4A), but also the integrity of these lysosomes is very high as determined by the very low percentage of hexosaminidase detected in integral lysosomes (leaking) vs detergent treated lysomes (Fig. S4B).

The authors show mis-localisation of Lamp-2a in various figures. However, upon inhibition of lysosomal degradation they state "...that defective expression and localization of LAMP2A in cystinotic cells is due to its excessive lysosomal degradation:" So obviously there is no mis-localisation, because otherwise the protein would not be accessible to lysosomal degradation.

We agree with the reviewer that this was not explained in detail. The reviewer is right as LAMP2A is routed to the lysosomes in order to be degraded by lysosomal proteases. However, our data suggest that only a fraction of total cellular LAMP2A is localized at lysosomes. By IF analysis LAMP2A is rarely detected in colocalization with LAMP1 in Ctns-/- cells (Red arrow in new Fig 5a). Instead, most LAMP2A is detected at structures different from those positive for LAMP1. These data, together with the observation that LAMP2A localization at

lysosomes increases in response to lysosomal protease inhibition suggest that those LAMP2A molecules that reach the lysosomal membrane are susceptible to rapid degradation. Similar behavior has been described for lysosomal membrane proteins with altered posttranslational modifications (Barriocanal et al *J Biol Chem* 261: 16755-16763,1986). We also show that LAMP2A has increased colocalization with Rab11 but decreased colocalization with VAMP7 in Ctns-/- cells suggesting that altered trafficking mechanisms take place in these cells. Again, this is also likely associated with structural changes that may differentially affect a subpopulations of LAMP2A molecules. Our data support that LAMP2A defects in Ctns-/- cells are not ruled by all-or-nothing defects; instead, they are caused by discrete alterations in LAMP2A subpopulations which is compatible with possible changes in posttranslational modifications. Supporting scenarios characterized by defects that affect only a subpopulation of cellular LAMP2 include TMEM165 deficiency, a congenital disorder of glycosylation where only a fraction of LAMP2 is affected (Foulquier et al, The American Journal of Human Genetics 91, 15–26, July 13, 2012). However, we are not necessarily saying that LAMP2A glycosylation is affected in cystinosis, as other posttranslational modifications have also been implicated in fine-tuning the sorting signals of lysosomal membrane proteins (Saftig P, Klumperman J (2009). Nat Rev Mol Cell Biol 10**:** 623-635). Rather, we argue that partial mislocalization of LAMP2A is a plausible scenario. This is now discussed in page 11, 1st paragraph, line 7 in the "Results" section and in Pages 14 and 15 "Discussion".

It may well be that Lamp-1 and Lamp-2a are partially segregated in the lysosomal membrane.

We appreciate the input and determined this experimentally using high resolution microscopy (Stochastic optical reconstruction microscopy (STORM)). Using this approach, LAMP2A was observed in close proximity (~10 nm, STORM resolution limit) to LAMP1 in wild type cells, suggesting that they are distributed at common lysosomal microdomains. However, in *Ctns*-/- cells, LAMP2A was mostly distributed at LAMP1-negative structures or in the proximity but not adjacent to LAMP1 as observed for wild type cells. Altogether confocal and STORM analyses suggest that in cystinotic cells LAMP2A is mainly present at non-lysosomal structures. In addition, in the lysosomal membrane of *Ctns*$_{-/-}$ cells, LAMP2A and LAMP1 appear partially segregated in distinct microdomains (new Fig. 5B).

The authors state several possibilities for the surprisingly selective effect of the protease inhibitor treatment, however to the reviewers knowledge there is no convincing mechanistic insight presented so far.

Based on previous studies, the selective degradation of LAMP2A in lysosomes is explained by the regulation of the dynamics between its multimeric and monomeric states, which is regulated by the binding of selective proteins including hsc70, glial fibrillary acidic protein (GFAP) and elongation factor 1 α (EF1α) to its unique carboxy-terminal cytosolic tail (Kauishik and Cuervo, Trends

in Cell Biology, Volume 22, August 2012, Pages 407–417). Also discussed in page 14, bottom paragraph “Discussion”.

In this context the reviewer likes to mention the critical importance of unequivocal detection of Lamp-2a as the predicted key player in CMA. The authors used a commercially available antibody against Lamp-2a, which was raised against a region of more than 50 residues. There is sequence variation within this region of the three Lamp-2a isoforms, however it remains unclear to me where the isoform specificity of the used antibody is documented.

We have now repeated experiments using an antibody raised against the 12 amino acid cytosolic tail of rat LAMP2A that is identical to that in mouse (GLKRHHTGYEQF) and was largely demonstrated to detect mouse LAMP2A but not other LAMP2 isoforms (Cuervo and Dice, science 1996; Cuervo and Dice Journal of Cell Science 113, 4441-4450 (2000). Both the anti-LAMP2A antibody (Ab18528) and the now included anti-12aa LAMP2A antibody recognize the expected LAMP2A band of ~95kDa and showed significant decreased expression in cystinotic lysosomes (new Fig. 4D).

Fig. 4: Assays for CMA: The figure shows the workflow for a lysosome preparation, however the quality of the preparation is not documented (see above).

We have included a new workflow now showing lysosomal quality control (New Fig. 8). The results of these QC experiments are included in new Supplementary Fig. S4

In B, a western is presented to document the CMA mediated degradation of GAPDH. In the full reaction (lane3), no remaining GAPDH is visible. However, the statistics presented in C document an average protease resistant fraction of around 40%. Thus, B shows a rather extreme experiment, but not a representative example??

We agree with the reviewer. Old Figure 4B (New Fig. 8B) now shows a more representative WB showing milder degradation of GAPDH.

I am puzzled by the figure legend, because the differences between assays in B and E are not indicated. Were lysosomes in B not separated by centrifugation? In E, the authors claim that the data shows GAPDH bound to the lysosomal membrane and the "internalized" fraction. The designation of this fraction is inappropriate, because the process is thought to occur via translocation through the membrane. Further, the controls are missing. The input amount of GAPDH is missing, as well as GAPDH incubated without lysoso
mes. For appropriate analysis, the authors would need to show the levels of GAPDH in the supernatant after centrifugation.

This is now clearly explained in the Figure legend (new Figure 8B). For CMA assays, Lysosomes were incubated with the CMA substrate GAPDH in the presence or absence of ATP (necessary for CMA) or protease inhibitors. A fraction of the total CMA reaction was analyzed by WB. GAPDH was analyzed by immunoblotting.

We have now included the analysis of LAMP1 as loading control (new Figure 8B). This figure also include the input amount of GAPDH incubated without lysosomes.

To avoid confusions and because old Figure 4E is not a key experiment for the conclusions of the manuscript, the uptake assays (old Figure 4E) was now removed.

The respective M&M section is unclear: What is the difference between a CMA assay and an "uptake assay"? Were lysosomes only pelleted for the latter assay?

The uptake assay has been removed from M&M.

As displayed, the data is insufficient to draw any conclusion and the interpretation of the data on page 10 is a massive over-interpretation.
We followed the reviewer's suggestions. All data and interpretations related to substrate binding and uptake have now been removed from the manuscript.

Discussion: The authors state defective degradation of CMA substrates, but they only analysed a single one.
"CMA substrates" has been changed to "the CMA substrate GAPDH" in the Discussion.

The authors speak about "...the decreased expression of LAMP2A at the lysosomal membrane", which is misleading. They probably mean the lysosomal content of Lamp-2a, or the detectable levels of the protein.

We have now removed "...the decreased expression of LAMP2A at the lysosomal membrane" and now refer to "decreased detectable levels of LAMP2A…"

Further in the text they present a „hypothesis" about a direct role of CTNS in the regulation of the „CMA receptor complex". This is not a hypothesis, but pure speculation.

We agree with the reviewer comment. This has been removed.

Finally, at the very end of the discussion the statements of the authors are redundant.
We agree with the reviewer comment. This has been modified.

In summary, I see massive deficits in the analysis of CMA and its interpretation. Furthermore, it remains unclear, whether mis-localisation of Lamp-2a occurs at all. Both issue require adequate attention in a revision of the manuscript.
We have followed all the reviewer's suggestions. We have included new controls for the CMA assay, new analyses using a different anti-LAMP2A antibody directed towards the 12aa of the unique cytosolic domain of mouse LAMP2A, included high resolution microscopy analysis of sub-domain distribution of LAMP2A, new IF analysis showing mislocalization of endogenous LAMP2A at trafficking vesicles. Eliminated binding/uptake assay and modified text to accommodate all the reviewer's suggestions.

We thanks the reviewers for their comments as we believe this is now a much stronger manuscript because of their suggestions.

2nd Editorial Decision 24 November 2014

Thank you for the submission of your revised manuscript to EMBO Molecular Medicine. I again apologise that it has taken longer than usual to get back to you on your manuscript. As you know, in this case we experienced unusual difficulties in obtaining the evaluations in a timely manner.

We have now received the enclosed reports from the Reviewers that were asked to re-assess it. As you will see the two Reviewers still require clarification on specific points.

Briefly, Reviewer 1 lists a number of issues pertaining to scholarship precision that require your action. Reviewer 2 is more reserved and mentions a number of remaining concerns with the quality and significance of your imaging and the over-reaching conclusions drawn from them. I agree with this Reviewer that the issues need to be solved before your manuscript can be considered further for publication.

Although I will not be asking you to provide further experimentation at this point, please 1) send me a rebuttal on the points raised and an additional copy of your manuscript with the changes made appropriately highlighted; 2) provide the data and improved images if available, or in alternative amend your text as to avoid overreaching conclusions. I am prepared in principle to make an Editorial decision on your final, revised version, provided the issues raised are dealt with as mentioned above.

Please also consider the following Editorial amendments/requests:

1) As per our Author Guidelines, the description of all reported data that includes statistical testing must state the name of the statistical test used to generate error bars and P values, the number (n) of independent experiments underlying each data point (not replicate measures of one sample), and the actual P value for each test (not merely 'significant' or 'P < 0.05').

2) The manuscript must include a statement in the Materials and Methods identifying the institutional and/or licensing committee approving the experiments, including any relevant details (like how many animals were used, of which gender, at what age, which strains, if genetically modified, on which background, housing details, etc). We encourage authors to follow the ARRIVE guidelines for reporting studies involving animals. Please see the EQUATOR website for details: http://www.equator-network.org/reporting-guidelines/improving-bioscience-research-reporting-the-arrive-guidelines-for-reporting-animal-research/

3) We are now encouraging the publication of source data, particularly for electrophoretic gels and blots, with the aim of making primary data more accessible and transparent to the reader. Would you be willing to provide a PDF file per figure that contains the original, uncropped and unprocessed scans of all or at least the key gels used in the manuscript? The PDF files should be labeled with the appropriate figure/panel number, and should have molecular weight markers; further annotation may be useful but is not essential. The PDF files will be published online with the article as supplementary "Source Data" files. If you have any questions regarding this just contact me.

4) Every published paper now includes a 'Synopsis' to further enhance discoverability. Synopses are displayed on the journal webpage and are freely accessible to all readers. They include a short standfirst (to be written by the editor) as well as 2-5 one sentence bullet points that summarise the paper (to be written by the author). Please provide the short list of bullet points that summarise the key NEW findings. The bullet points should be designed to be complementary to the abstract - i.e. not repeat the same text. We encourage inclusion of key acronyms and quantitative information. Please use the passive voice. Please attach these in a separate file or send them by email, we will incorporate them accordingly.

I look forward to reading a new revised version of your manuscript as soon as possible.

***** Reviewer's comments *****

Referee #1 (Comments on Novelty/Model System):

The revision has greatly improved the manuscript and my comments have been addressed.
The results are relevant since they help in clarifying the pathology of a human disease.

Referee #1 (Remarks):

The revision has greatly improved the manuscript and my comments have been addressed.

There are some minor points to address:

1. In Abstract the authors still state: "in CTNS-deficient cells and degradation of CMA substrates are defective", although they only tested one CMA substrate (GAPDH).

2. On page 15, the authors write "LC3B-II, a protein known to specifically associate with autophagosomes and not with other vesicular structures"
This is generally correct, however, LC3 can also associate with phagosomes in some cell types and specific conditions (LC3 assisted phagocytosis, LAP). PLoS Pathog. 2014 Jun 5;10(6):e1004159

3. The authors now use the spelling LAMP2A in the text, but in the figures they still use the old spelling LAMP2a. This needs attention.

4. Page 16: "...the sorting signals and stability of lysosomal membrane proteins (Saftig & Klumperman,2009) and (Barriocanal et al, 1986), respectively. ER initiated post-translational modification defects in cystinosis are suggested by previous works showing high levels of ER stress and expansion in cystinotic cells"

Better: "...the sorting signals and stability of lysosomal membrane proteins, respectively (Saftig & Klumperman,2009; Barriocanal et al, 1986). Defects in ER initiated post-translational modifications in cystinosis are suggested by previous studies, showing high levels of ER stress and expansion in cystinotic cells"

Referee #3 (Remarks):

The revised version of the manuscript has greatly improved, although certain aspects still require attention and re-wording:

Legend to Fig. 4: It is incorrect to "... analyse LAMP2A expression.." in lysosomes. One can probe the content, or the amounts of it.

Fig. 5B: What should STORM microscopy show? If two antigens do not colocalize in confocal images - they will never do so when using super resolution microscopy. Moreover, quite a number of lysosomes of control cells are resolved to rings in the confocal images, but only to simple larger or smaller dots in STORM. One would expect that even small dots of 200nm should be resolved to rings in STORM. Thus, the overall quality of the analysis is questionable and therefore its application was useless as no further insight is gained. The respective main text (p.9) is unclear as well. What is the difference between "in proximity" and "adjacent". Examples for both are seen in the CTNS -/- cells!

Fig.6: This figure shows 6 images of LAMP2A in control and CTNS -/- fibroblasts. It is surprising how remarkably different this staining can be. The LAMP2A staining of CTNS -/- cells stained also for Vamp7 look completely different from those stained for Rab11, which exhibit a strong nuclear background/non-specific staining??

The figure further shows staining of the ER, but it remains unclear whether the KDEL receptor was stained, or whether a KDEL-reporter construct was used. Unfortunately, non of the organelle stainings is without doubt. There are numerous marker proteins of the ER that are used in many publications to stain the ER (BiP, PDI, Calnexin, Calreticulin), while the ER staining here is faint and more indicative of Golgi distribution.

Vamp7 is well known to be distributed throughout the endosomal system, mediating fusion between subcompartments, with lysosomes and the plasma membrane. Due to its relatively low abundance its staining is usually faint with dots dispersed throughout the cytoplasm. The image for Vamp7 in the control cells is thus rather unusual.

The same is true for the staining of Rab11. Again, in most cells staining of Rab GTPases is not easy and in fact other markers of recycling endosomes such as subunits of Retromer, endocytosed Tf/TfR or other rock-solid markers of the endomembrane system such as EEA1 or MPR46/300 appear much more appropriate.

From what the authors show, LAMP2A may indeed be segregated from its normal subcellular localisation in the knockout cells, however it still remains rather vague to which membrane it is re-directed.

In the main text the authors mention a single reference for Vamp7 (Pol et al., 2013), which is the one and so far only report on a Vamp7 function in transport from the Golgi to late endosomes. Other references that complement each other and show its well-established function in endosomes/lysosomes are not mentioned or discussed.

Based on a single image (localisation of Vamp7/LAMP2A) the authors suggest a defective Vamp-7 dependent transport system (page 15). This is pure speculation and an over-interpretation of a simple localisation study. Without any functional assays, without any deeper insight into a mechanism the authors should be very carful with their statements and conclusions.

2nd Revision - authors' response 07 December 2014

Answers to Reviewer's comments

Referee #1 (Comments on Novelty/Model System):

The revision has greatly improved the manuscript and my comments have been addressed.
The results are relevant since they help in clarifying the pathology of a human disease.

Referee #1 (Remarks):

The revision has greatly improved the manuscript and my comments have been addressed.

There are some minor points to address:

1. In Abstract the authors still state: "in CTNS-deficient cells and degradation of CMA substrates are defective", although they only tested one CMA substrate (GAPDH).
This is now corrected.

2. On page 15, the authors write "LC3B-II, a protein known to specifically associate with autophagosomes and not with other vesicular structures"
This is generally correct, however, LC3 can also associate with phagosomes in some cell types and specific conditions (LC3 assisted phagocytosis, LAP). PLoS Pathog. 2014 Jun 5;10(6):e1004159
This is now corrected. We wrote: "LC3B-II, a protein known to specifically associate with autophagosomes and rarely with other vesicular structures"

3. The authors now use the spelling LAMP2A in the text, but in the figures they still use the old spelling LAMP2a. This needs attention.
This is now corrected.

4. Page 16: "...the sorting signals and stability of lysosomal membrane proteins (Saftig & Klumperman,2009) and (Barriocanal et al, 1986), respectively. ER initiated post-translational modification defects in cystinosis are suggested by previous works showing high levels of ER stress and expansion in cystinotic cells"

Better: "...the sorting signals and stability of lysosomal membrane proteins, respectively (Saftig & Klumperman,2009; Barriocanal et al, 1986). Defects in ER initiated post-translational modifications in cystinosis are suggested by previous studies, showing high levels of ER stress and expansion in cystinotic cells"
This is now corrected in Page 16, top paragraph.

Referee #3 (Remarks):

The revised version of the manuscript has greatly improved, although certain aspects still require attention and re-wording:

Legend to Fig. 4: It is incorrect to "... analyse LAMP2A expression.." in lysosomes. One can probe the content, or the amounts of it.

This is now corrected. We wrote: “D, Comparative analysis of the amount of LAMP2A in wild type or *Ctns*$^{-/-}$ liver lysosomes……….”

Fig. 5B: What should STORM microscopy show? If two antigens do not colocalize in confocal images - they will never do so when using super resolution microscopy. STORM shows high resolution images of the distribution of proteins under analysis at single molecule resolution. This analysis helped to answer a point raised by the reviewer: Are LAMP1 and LAMP2a in different subdomains? Our STORM analysis shows that in wild type cells LAMP1 and LAMP2a are distributed at similar subdomains of the lysosomal membranes. As for CTNS-KO cells, most LAMP2A single molecules are separated from LAMP1 either at different subdomains or at different structures. Although STORM would not differentiate between this two possible scenarios, this was largely addressed by our confocal studies showing that most but not all LAMP2A is mislocalized in these cells.

Moreover, quite a number of lysosomes of control cells are resolved to rings in the confocal images, but only to simple larger or smaller dots in STORM. One would expect that even small dots of 200nm should be resolved to rings in STORM. STORM images would resolve into rings only if the proteins in the analysis occupy a continuum space in the thickness of the sample and if the distance between these molecules in that x,y plane (lateral resolution) is around 20 nm (or up to 50 nm when antibodies are utilized for detection). While this is always the case for molecules like alpha-tubulin or polymerized actin, this is not the case for LAMP1, which occupies subdomains in the lysosomal membrane separated by lipids and other proteins. Thus, the overall quality of the analysis is questionable and therefore its application was useless as no further insight is gained. The insight gained includes: LAMP1 and LAMP2A occupy the same subdomains in wild type cells (to the best of our knowledge this was not shown before). Also, the reduced lysosomal LAMP2A in KO cells may not be in the same subdomain as LAMP1 as deducted from images showing LAMP2A close to but not adjacent (not within the 20-50 nm distance limit) to LAMP1. The respective main text (p.9) is unclear as well. What is the difference between "in proximity" and "adjacent". The resolution of STORM is around 20 nm (or up to 50 nm when antibodies are utilized for detection). In proximity refers to two molecules that are separated for more than 50 nm and therefore do not appear in contact with each other. Adjacent refers to two molecules that are at 20-50 nm distance from each other and appear in contact with each other. This is now explained more clearly in the text (Page 10, top paragraph). Examples for both are seen in the CTNS -/- cells! This is expected; however, the phenotype of adjacent (in contact, 10-50 nm) LAMP1-LAMP2A molecules is greater in wild type cells than in cystinosis.

Fig.6: This figure shows 6 images of LAMP2A in control and CTNS -/- fibroblasts. It is surprising how remarkably different this staining can be. The LAMP2A staining of CTNS -/- cells stained also for Vamp7 look completely different from those stained for Rab11, which exhibit a strong nuclear background/non-specific staining??

Improved images were now provided for the Rab11/LAMP2A staining.

The figure further shows staining of the ER, but it remains unclear whether the KDEL receptor was stained, or whether a KDEL-reporter construct was used. Unfortunately, non of the organelle stainings is without doubt. There are numerous marker proteins of the ER that are used in many publications to stain the ER (BiP, PDI, Calnexin, Calreticulin), while the ER staining here is faint and more indicative of Golgi distribution.

Improved images were now provided for KDEL staining in wild type cells. In these experiments, we stained the ER using a largely validated antibody that recognizes the KDEL sequence in endogenous Grp78 and Grp94 (anti-Grp78/94 (Assay Designs). We renamed the images with the name of the

endogenous proteins to avoid confusion. The staining is more reticular in the WT cells. The antibody heavily labeled large structures in CTNS-KO cells (now indicated with arrows). As shown previously by our group, these structures correspond to expanded ER (determined by electron microscopy) due to high levels of ER stress in these cells (Johnson et al Mol Biol Cell 2013).
Vamp7 is well known to be distributed throughout the endosomal system, mediating fusion between subcompartments, with lysosomes and the plasma membrane. Due to its relatively low abundance its staining is usually faint with dots dispersed throughout the cytoplasm. The image for Vamp7 in the control cells is thus rather unusual.
The staining of endogenous VAMP7 shown here is the expected of a protein that is distributed throughout the endosomal system and is similar to that shown by others (see, for example, Fraldi et al. The EMBO Journal (2010) 29, 3607–3620). This is now indicated in page 10 last paragraph.
The same is true for the staining of Rab11. Again, in most cells staining of Rab GTPases is not easy and in fact other markers of recycling endosomes such as subunits of Retromer, endocytosed Tf/TfR or other rock-solid markers of the endomembrane system such as EEA1 or MPR46/300 appear much more appropriate.
Although we agree with the reviewer that the staining of Rab GTPases could be challenging, the staining for endogenous Rab11 worked quite well in our hands using the protocol described under Material and Methods.

From what the authors show, LAMP2A may indeed be segregated from its normal subcellular localisation in the knockout cells, however it still remains rather vague to which membrane it is re-directed.
By showing increased colocalization with Rab11 and reduced colocalization with VAMP7 our data is starting to elucidate the nature of these membranes.
In the main text the authors mention a single reference for Vamp7 (Pol et al., 2013), which is the one and so far only report on a Vamp7 function in transport from the Golgi to late endosomes. Other references that complement each other and show its well-established function in endosomes/lysosomes are not mentioned or discussed.
Other references have now been added (Page 10, last paragraph and Page 11 first paragraph).
Based on a single image (localisation of Vamp7/LAMP2A) the authors suggest a defective Vamp-7 dependent transport system (page 15). This is pure speculation and an over-interpretation of a simple localisation study. Without any functional assays, without any deeper insight into a mechanism the authors should be very carful with their statements and conclusions.
The reviewer is right and our statements have been now revised (Page 15, last paragraph).
